# A Review of Failure Modes and Safety Strategies of Lithium‐Ion Batteries from Materials to Systems

**DOI:** 10.1002/advs.76228

**Published:** 2026-06-30

**Authors:** Jin Hyeok Yang, Jae Yoon Sung, Hyunji Kweon, Seohyun Kim, Byunghoon Kim, Jongsoon Kim, Junyoung Mun, Jung Ho Kim, Ki Jae Kim

**Affiliations:** ^1^ Department of Energy Science Sungkyunkwan University (SKKU) Suwon Republic of Korea; ^2^ Department of Future Energy Engineering Sungkyunkwan University (SKKU) Suwon Republic of Korea; ^3^ School of Advanced Materials Science and Engineering Sungkyunkwan University (SKKU) Suwon Republic of Korea; ^4^ SKKU Institute of Energy Science and Technology (SIEST) Sungkyunkwan University (SKKU) Suwon Republic of Korea; ^5^ Institute For Superconducting and Electronic Materials (ISEM) University of Wollongong North Wollongong New South Wales Australia

**Keywords:** cascade failure, intrinsic safety, lithium‐ion battery, multiphysics coupling, safety engineering, thermal runaway

## Abstract

Lithium‐ion batteries (LIBs) have rapidly proliferated due to their high energy density, fast charging capability, and long cycle life. However, intrinsic thermal instability poses serious safety concerns, as failures can trigger fires or explosions, hindering large‐scale deployment in electric vehicles and energy storage systems. This review examines thermal runaway mechanisms of LIBs by dividing them into sequential stages involving anode–electrolyte interface breakdown, separator shrinkage, electrolyte decomposition, and cathode oxygen release, and by analyzing how multiphysical couplings among thermal, electrochemical, mechanical, and chemical factors govern cascade evolution. Furthermore, the review explores safety enhancement strategies across multiple scales, ranging from material‐level improvements such as surface coating and doping of Ni‐rich cathodes, ceramic‐based separators, and non‐flammable electrolyte additives, to structural designs of cells, modules, and packs, as well as system‐level diagnostics and control frameworks utilizing battery management systems, artificial intelligence, and digital twin–based prediction models. Addressing the increasing risks associated with LIB applications remains a critical challenge in battery and fire safety engineering. This review therefore proposes a cascade‐aware safety framework that links quantitative, stage‐specific failure indicators to material‐, cell‐, module‐, pack‐ and system‐level interventions for intrinsic safety design in high‐energy‐density LIBs.

## Graphical abstract

A cascade‐aware framework is presented for lithium‐ion battery safety, linking thermal runaway initiation, acceleration, runaway reaction, and propagation with material‐, cell/pack‐, and system‐level interventions. By integrating failure mechanisms, quantitative safety indicators, and staged interception strategies, this review highlights how safer high‐energy batteries can be designed from reactive components to intelligent battery systems.

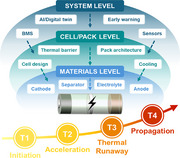

## 1 Introduction

### 1.1 Overview of Lithium‐Ion Batteries

Fossil fuels have powered global infrastructure over a century, including transportation, manufacturing, and residential power systems, and still supply more than 80% of total energy demand [1]. Their continued use has accelerated climate change, air pollution, and resource depletion, and has spurred the transition toward low‐carbon energy systems [2]. Electrochemical energy storage, the most prominent type of lithium‐ion batteries (LIBs), is central to this shift [3]. LIBs have become indispensable owing to their high energy density, long cycle life, and high operating voltage. Initially confined to portable electronics, they now dominate large‐scale applications such as electric vehicles (EVs) and stationary energy storage systems (ESS) [4, 5, 6]. Growing deployment has intensified performance demands [5], driving research into high‐capacity electrodes (e.g., silicon anodes and nickel (Ni)‐rich cathodes), novel electrolytes, and advanced manufacturing [7, 8, 9, 10].

However, increasing the energy density of LIBs often comes at the cost of heightened safety risks [11, 12]. Flammable electrolytes, oxygen‐releasing cathodes, and lithium plating make LIBs intrinsically prone to thermal runaway [13, 14, 15]. Moreover, battery failures rarely stem from a single factor but from coupled electrical, mechanical, thermal runaway, and manufacturing stresses that cascade into exothermic reactions [16, 17, 18]. Large‐format batteries, such as pouch or cylinder‐type cells, packs, and modules, further magnify these risks because localized faults can propagate rapidly [19, 20]. Achieving higher energy density requires parallel advances in diagnostics, protective architectures, and system‐level safety designs [21]. Understanding these hazards is critical not only for optimizing current devices, but also for enabling next‐generation batteries with high performance and reliability [22]. Addressing this challenge requires a comprehensive understanding of LIB failure mechanisms and a systematic evaluation of existing and emerging mitigation strategies [17].

This review frames LIB safety in three dimensions: (i) lessons from representative failure incidents [23, 24], (ii) mechanistic pathways of thermal runaway, and (iii) critical assessments of mitigation strategies that span materials, architectures, and system controls [25, 26, 27]. Collectively, these perspectives outline the current state of the science behind LIB safety, vulnerabilities, and priorities for safer energy storage technologies [28, 29, 30, 31]. In line with this scope, the examples discussed in each section were chosen to illustrate representative failure pathways and safety strategies across material, cell, module, pack, and system levels.

### 1.2 Representative Safety Incidents and Their Impact

Although LIBs are generally reliable under standard operating conditions, they are highly sensitive to excursions beyond their electrochemical or mechanical limits [24], and failures remain statistically rare; however, real‐world incidents have underscored their consequences for consumer electronics, EVs, aviation equipment, and ESS [24, 32]. Despite the integration of passive and active safeguards, field data revealed that these measures do not always avert hazardous outcomes, particularly in large‐scale or densely packed systems [33, 34]. Several high‐profile accidents involving consumer electronics, electric vehicles, aviation, and stationary storage have underscored the multifaceted risks associated with LIB failure [24, 32]. These events are attributed to manufacturing defects, inadequate thermal dissipation, mechanical damage, and flawed system integration. Table 1 categorizes the representative LIB failures by application sector, outlining the underlying causes and associated consequences in practical contexts.

**TABLE 1:** Representative LIB fire and explosion incidents across diverse sectors.

Sector	Year	Incident	Cause	Consequences	References
ESS	2019	APS McMicken BESS Fire & Explosion	Internal cell failure → thermal runaway & gas deflagration	Container destroyed	[35]
ESS	2021	Victorian Big Battery (Tesla Megapack) Fire	Coolant leak → arcing → thermal runaway	2 packs lost, ∼3‑day fire	[36]
ESS	2021	Beijing Dahongmen BESS Explosion	Overheating / thermal runaway propagation during firefighting	Infrastructural damage	[37]
ESS	2022	Elkhorn BESS (Moss Landing) Fire	Rack fault → thermal event; water interaction	System shutdown & repairs	[38]
ESS	2017‐2019	23 BESS Fires (South Korea)	Installation/BMS/PCS defects + environmental factors	≈$32 M losses; 522 units halted	[39]
ESS	2025	Vistra Moss Landing BESS Fire	Cause under investigation (large‑scale Li‑ion fire, HF gas concerns)	∼80% of 300 MW array damaged; evacuation	[40]
EV	2018	Tesla, BEV (Chongqing, China)	Fire in the parked vehicle (Spontaneous ignition)	Fire spread in 10 s, igniting 3 cars	[41]
EV	2018	Porsche Panamera, PHEV (Bangkok, Thailand)	Caught fire during charging	Unsafe indoor charging caused house fire	[42]
EV	2018	Tesla Model X, BEV (California, USA)	Post‐crash fire	Fire reoccurred twice after 5 days	[43]
EV	2018	Tesla Model S, BEV (California, USA)	Caught fire during driving	Fire extinguished with no reoccurrence	[44]
EV	2021	Tesla Model S, P100D (Texas, USA)	Post‐crash fire	Crash damaged battery, causing fire	[45]
EV	2023	Mercedes‐Benz EQE (Seoul, Korea)	Parked vehicle caught fire (Spontaneous ignition)	The garage explosion damaged 880 vehicles	[46]
EV	2017	Tesla model X SUV (California, USA)	Post‐crash fire	Fire spread from SUV to garage and house	[47]
Mobile	2025	Motorola Moto E32 (Anapolis Brazil)	The phone exploded in back pocket (Spontaneous ignition)	2nd–3rd degree burns; hair/clothes burned, trauma	[48]
Mobile	2016	Samsung Galaxy Note 7 (Global Recall)	Battery design and manufacturing defects (electrode short‐circuit, poor welding)	2.5M recalled; fires, explosions, flight ban, brand damage	[49]
Mobile	2016	iPhone (Gareth Clear, Australia)	Physical impact from minor fall → lithium‐ion battery damage	3rd‐degree thigh burns, skin graft surgery	[50]
Mobile	2016	iPhone 6 (Alaska Airlines flight)	The phone exploded (Spontaneous ignition)	Passenger panic, emergency landing delay	[51]
Other	2024	Laptop Battery (Faisalabad, Pakistan)	Caught fire during charging (thermal runaway)	2 deaths; 7 injured; house destroyed	[52]
Other	2025	iStore's Power Banks (Global Recall)	Caught fire due to overheating during charging	Minor burn, $15K in damage	[53]
Other	2024	Electric bike (Sydney, Australia)	Caught fire during charging (thermal runaway)	7 treated for smoke, 100 evacuated	[54, 55]
Other	2022	Electirc scooter (Queensland, Australia)	Caught fire due to being connected to an incompatible charger	Explosion and fire caused by thermal runaway	[56]

The widespread deployment of LIBs, including their applications in energy storage, electric mobility, and portable electronics, has introduced safety challenges rooted in material‐level reactivity and system‐level vulnerabilities [57, 58]. These risks are not dictated by material properties alone but emerge from the interplay of design architecture, operating conditions, control strategies, and manufacturing consistency [59, 60]. Failures can be initiated by diverse triggers such as thermal runaway from overcharge, external heating, mechanical stress, latent cell defects, or inadequate protection at the module and system levels [18, 61, 62]. The failure consequences have been severe, encompassing human injuries, property loss, large‐scale recalls, and regulatory actions [63, 64]. As the energy density and system complexity increases, conventional passive safeguards are unlikely to fully prevent fault propagation. The following section examines the mechanisms of thermal runaway and associated failure modes in LIBs [62].

## 2 Factors Causing Failures in LIBs

**FIGURE 1:**
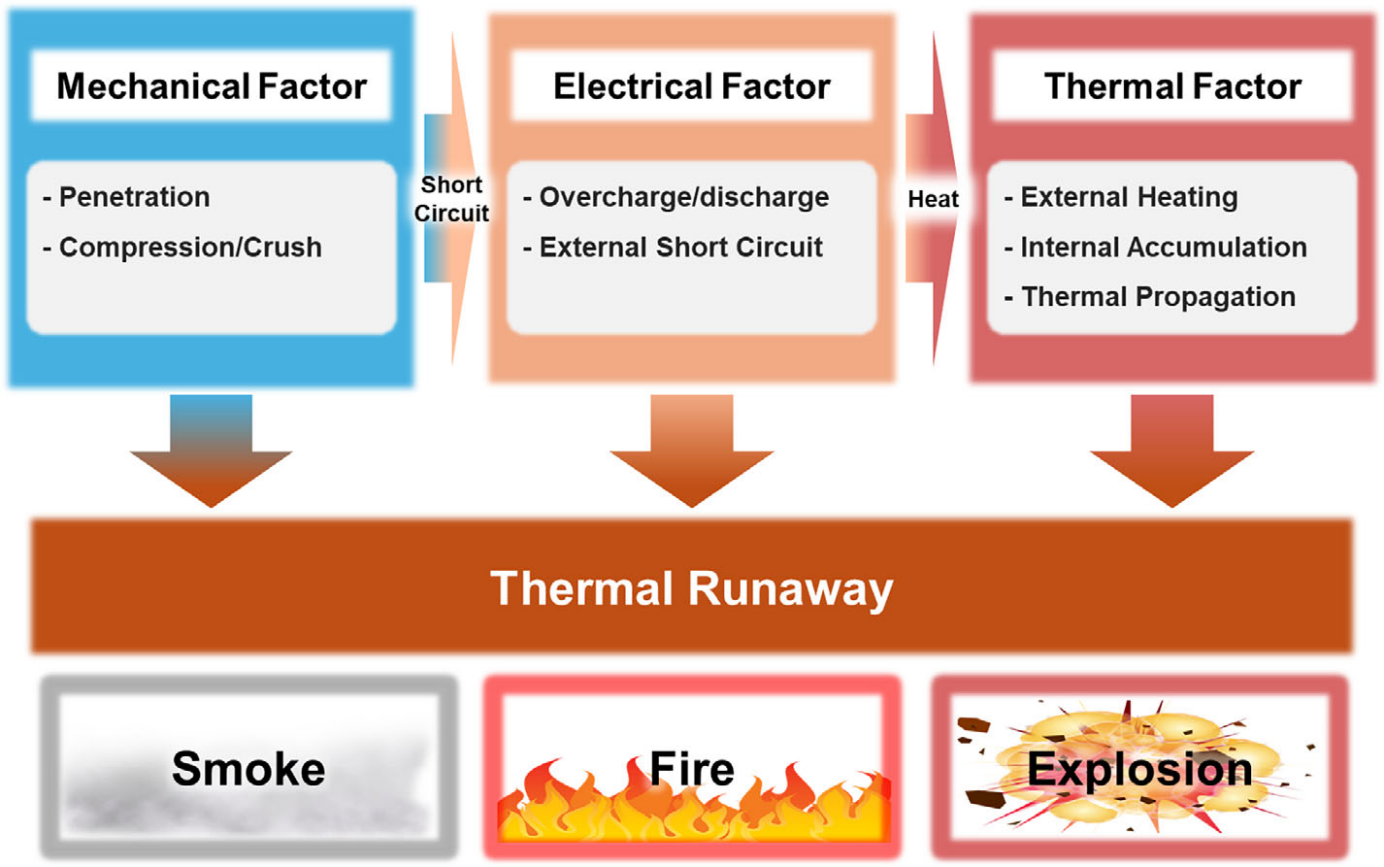
Key triggering factors for thermal runaway in LIBs. Mechanical, electrical, and thermal abuses initiate internal short circuits and heat accumulation, leading to progressive reactions that manifest as smoke, fire, or explosion.

As high‐energy storage devices, LIBs are inherently prone to cascading failures; even minor abnormalities can trigger heat generation and chemical reactions, leading to a rapid temperature rise and thermal runaway [16, 65]. These triggers are broadly categorized into three classes: mechanical, electrical, and thermal, each of which initiates a distinct failure pathway (Figure 1). This section discusses the triggers, categorized by their origin and failure mechanisms.

### 2.1 Mechanical Abuse

Mechanical factors refer to externally applied physical stresses that result in the structural deformation or displacement of LIB cells. Common forms of mechanical abuse, such as penetration, compression or crushing, are frequently implicated in failure events. Mechanical factors arise from externally applied stresses that deform or displace internal cell components. Penetration, compression, and crushing are the most frequent and hazardous forms of abuse, often compromising the integrity of the internal components and initiating short circuits that can cascade into thermal runaway [66, 67].

#### 2.1.1 Penetration

Penetration is one of the most direct and severe types of mechanical insults [66]. Unlike compression or crushing, which usually involves progressive deformation, penetration can instantly breach the separator, connecting the anode and cathode, causing localized internal short circuits (ISCs). This triggers rapid local Joule heating followed by electrolyte decomposition, gas release, and potentially catastrophic runaway. High‐speed X‐ray radiography by Yokoshima et al. provided the first real‐time visualization of nail penetration failure, showing a stepwise progression: localized current surges melting the nail tip, electrolyte boiling, rapid gas expansion, and electrode collapse [68]. Notably, early‐stage short circuits are intermittent, producing a spatially localized, nonlinear heat distribution. Chen et al. systematically studied high‐energy 21700 cells (≈257 Wh kg^−1^) under nail penetration, varying state of charge (SOC), nail size, depth, speed, and entry location [69]. Using X‐ray computed tomography (CT) before and after abuse, they quantified the internal structural damage, traced the ejection pathways of metal fragments from the current collector, and measured gas accumulation. Coupled electro‐thermal‐mechanical analysis showed that the rupture was governed by (i) internal pressure surge, (ii) mechanical strength of the can, and (iii) effective vent/rupture path area. Additionally, thermal runaway escalation depended more on SOC‐dependent gas generation and confinement‐induced pressure than on the initial ISC.

From a modeling perspective, Ren et al. developed a multilayer ISC simulation to evaluate the evolving thermal‑runaway risk during nail penetration [70]. The framework models the cell as stacked electrochemical units and couples the electrochemical, thermal, and ISC behaviors consistent with multiscale modeling strategies. Simulations revealed sequential voltage drops across the penetrated layers and the dynamic redistribution of short‐circuit currents, altering the local heat generation and accumulation. High‐temperature hot spots originate where the separator failure initiates Joule heating and exothermic side reactions, which intensify with higher SOC [70]. Zhao et al. presented a nailpenetration model for large‐format cells, with parametric studies showing that slower penetration speeds and smaller nail diameters exacerbate thermal severity by prolonging the heat input and concentrating it within a narrower region. These findings underscore the importance of safety testing protocols and design optimizations considering the combined effects of the cell geometry and abuse rate [71].

#### 2.1.2 Compression and Crush

Compression and crushing are among the most common and high‐risk mechanical abuses of LIBs and act as critical triggers for thermal runaway. These stresses occur when batteries experience direct external forces or structural deformations such as vehicle collisions, accidental drops, or excessive compression during assembly [72]. Hahn et al. systematically investigated the influence of varying the external clamping pressure on the thermal runaway behavior of pouch‐type cells [73]. In a quasi‐static crush test, four clamping pressures (0.05–0.5 MPa) were applied and compared with uncompressed controls. The results showed that lower clamping pressures extended the onset time for thermal runaway by up to 19%, whereas combustible‐gas volume and concentration increased by up to 80% in uncompressed cells. Crush‑type mechanical abuse induces out‑of‑plane compression that fractures separators, which in turn triggers ISCs and precipitates thermal runaway. Hahn et al. further demonstrated that reducing clamping pressure from 0.50 to 0 MPa delayed thermal runaway onset by ≈19% while nearly doubling combustible‐gas release, underscoring a trade‐off between initiation delay and gas hazard [73]. Quasi‐static tests and finite‐element models indicate that crush tolerance depends on cell geometry and stacking orientation, with simulations predicting separator tearing within ±8% of measured strain [74, 75]. Material studies further revealed that Si‐rich anodes improve crush strength but amplify exothermic reactions upon failure [64].

By integrating real‐time diagnostics, Jantscher et al. embedded an electrical circuit surrogate within the finite‐element stress model of an 18650 cell [76]. This approach flagged short‐circuit hotspots within approximately 2 ms of crush initiation and streamed the predicted voltage collapse and local temperatures, allowing virtual sensors to monitor runaway precursors. Beaumont et al. developed a macroscale orthotropic pouch‐cell model that reproduces experimental force‐displacement and internal strain fields [77]. The homogenized mesh allows the solver to run rapidly, enabling real‐time input to a digital twin observer and demonstrating how crash simulations can inform onboard diagnostics during pack design iterations. Recently, Kociu et al. incorporated layer‐resolved thermo‐electro‐mechanical elements and a 1 kHz Kalman‐filter estimator to capture both mechanical collapse and heat buildup. Their high‑fidelity model produced virtual thermocouple outputs in real time, enabling rapid “what‑if” assessments of the complete‐vehicle crush scenarios [78].

These studies show that compression and crushing are not merely mechanical stressors but also initiators of complex failure pathways, where structural deformation, localized heating, ISCs, and gas evolution converge can manifest. Accurate replication through controlled experiments, multi‐physics modeling, and real‐time diagnostic integration, including acoustic emission analysis, is essential for understanding the onset and progression of thermal runaway under realistic abuse conditions.

### 2.2 Electrical Factors

Electrical factors include abnormal charge flow, current imbalance, and circuit failure, which accelerate internal reactions and thermally destabilize LIBs. Although latent, these events are critical initiators of thermal runaway. Overcharge/discharge and external short circuits are the most common and sequential modes, respectively.

#### 2.2.1 Overcharge and Overdischarge

Electrical abuse, such as overcharge or overdischarge, disrupts electrochemical stability and accelerates degradation. Overcharge forces lithium insertion beyond the anode capacity, causing lithium plating, Solid Electrolyte Interphase (SEI) decomposition, and gas evolution [79]. Conversely, overdischarge can lower the potential of the copper current‐collector to below its stability limit, triggering copper dissolution, dendrite formation, and potential ISC [80, 81]. Both conditions promote abnormal energy accumulation and structural stress, thereby increasing the likelihood of thermal runaway.

Mao et al. monitored 18650 LiNi_0.6_Mn_0.2_Co_0.2_O_2_ (NMC622)/graphite cells beyond 170% SOC and identified four sequential stages: (i) cathode phase transformation with lattice‐oxygen release, (ii) lithium plating fracturing the SEI, (iii) electrolyte decomposition generating CO_2_/CO/H_2_, and (iv) the cells can rupture followed ≈20 s later by a >200 °C core‐temperature surge indicating full runaway [82]. Finegan et al. visualized the same sequence via high‐speed synchrotron X‐ray tomography, revealing a microcrack propagation, gas‐bubble growth, and >100 °C local hot spots migrating through the jelly‐roll, confirming that oxygen‐solvent reactions and Joule heating co‐localize to accelerate failure propagation [83]. Guo et al. showed that deep overdischarge (< 0 V) raises anode potential above the Cu redox window, and that dissolved Cu redeposits as dendrites that bridge approximately 80 µm to the cathode within 30 min, triggering thermal runaway at ∼140 °C [84]. A distinct voltage plateau at −12% SOC signaled the onset of Cu dissolution, emphasizing that collector corrosion, rather than simple Li depletion, is the dominant trigger in over‐discharge runaway. Collectively, these keystone studies reveal that although overcharge and over‐discharge arise from opposite electrochemical extremes, both converge on the same hazards: rapid self‐heating, flammable gas accumulation, and low‐impedance internal shorts, underscoring the need for real‐time SOC, voltage, and impedance surveillance in advanced battery‐management systems.

Building on these material‐level insights, Du et al. demonstrated that the structural heterogeneity within electrode materials plays a decisive role in dictating the initiation and propagation of thermal runaway under overcharge conditions [85]. Using a combination of in situ infrared thermography and multi‐physics simulations, they showed that regions of reduced porosity or uneven electrode thickness serve as localized hot spots where the current density and thermal flux are concentrated. Such irregularities accelerate the exothermic side reactions, including electrolyte decomposition and transition metal reduction, at specific sites within the cell. Notably, cells with pronounced heterogeneity consistently exhibited an earlier onset of runaway, higher peak temperatures, and more catastrophic structural collapse than their more uniform counterparts. Extending this perspective to mechanical responses, Xu et al. systematically examined the deformation of cells under overcharge‐induced thermal stress [86]. Their measurements revealed a pronounced internal expansion, leading to casing bulging and interlayer delamination. Pressure mapping and high‐resolution displacement tracking further indicated that stress accumulates preferentially near the electrode–separator interface, amplifying local thermal gradients and undermining cell integrity. Chen et al. highlighted an additional pathway, showing that the failure of the shell insulation can directly trigger ISCs [87]. In experiments on LiFePO_4_‐based cylindrical cells, the deliberate weakening of the insulation layer produced hotspots at the jelly roll edge, rapidly inducing separator breakdown and electrolyte decomposition. Within 90 s, these events cascade into a runaway reaction that breaches the casing. Detailed diagnostics confirmed that the critical temperature threshold was reached not only because of electrochemical heat release, but also because impaired insulation permitted localized heat accumulation without adequate dissipation.

Collectively, these studies underscore the fact that overcharge and overdischarge do not immediately yield to catastrophic failure. Instead, it initiates a sequence of coupled electrochemical, thermal, and mechanical degradations, which ultimately cause thermal runaway. As lithium‐ion technologies scale toward higher energy density and system complexities, identifying and disrupting this sequence at its earliest stages through both material design and embedded diagnostics will be central to achieving inherently safer battery systems.

#### 2.2.2 External Short Circuit

Unlike internal faults, external short circuits (ESCs) can occur without a structural breach of the cell, and are typically triggered by terminal contact failures, damaged insulation, or accidental bridging in high‐voltage pack systems [88]. When ESC occurs at a high SOC, the short‐circuit current reaches several hundred amperes within milliseconds, thereby producing severe localized heating. The rapid temperature rise not only accelerates electrolyte decomposition and gas evolution, but can also propagate exothermic reactions, culminating in thermal runaway if not promptly interrupted [89, 90].

The cell geometry and material selection is highly sensitive to the impact of ESCs. An et al. reported that cylindrical olivine LiFePO_4_ (LFP) cells subjected to ESC conditions below 50 mΩ resistance experienced temperature surges above 200 °C within 30 s. These thermal excursions were accompanied by structural deformations near the current collector tabs, highlighting the tendency for heat localization in highly conductive regions. Moreover, sequential ESC events in the same cell shortened the time to failure in subsequent tests, suggesting that residual internal damage compromises the thermal resilience and transforms ESCs from transient electrical disturbances into cumulative degradation pathways that integrate electrical, thermal, and structural stresses [91]. Zhang et al. compared conventional liquid electrolyte LIBs with solid/liquid hybrid electrolyte cells to examine how the internal architecture governs the thermal response under ESC conditions [92]. Although hybrid cells exhibited enhanced stability under normal cycling, they heated ∼40% faster and reached peak temperatures ∼80 °C higher during short‐circuit events, attributed to higher interfacial resistance and restricted ionic mobility in the composite electrolyte. Short circuits located near the current‐collector tabs further amplify the thermal escalation owing to the concentrated current flux and minimal path resistance.

Collectively, these findings highlight that the ESC hazards in LIBs cannot be reduced to electrical considerations alone. Instead, they emerge from a coupled interplay among the current distribution, cell architecture, heat dissipation, and system‐level controls. Therefore, effective mitigation requires advances in materials and mechanical design, complemented by integrated diagnostics, predictive thermal modeling, and circuit‐level fault management.

### 2.3 Thermal Factors

Thermal factors in LIBs refer to scenarios where temperature rises, whether externally imposed or internally generated, directly compromise stability and precipitate thermal runaway. Unlike electrical or mechanical triggers, thermal abuse can act independently or synergistically to accelerate decomposition reactions, gas evolution, and heat accumulation [93]. Although such effects often develop gradually, once critical thresholds are breached, they can escalate abruptly. The key risk scenarios include direct external heating, internal heat accumulation owing to insufficient dissipation, and inter‐cell propagation within densely packed modules [94].

#### 2.3.1 External Heating

Externally applied heat is a primary concern when assessing battery behavior under fire exposure, faulty thermal management, or ambient overheating. Wu et al. systematically studied commercial LiCoO_2_/graphite soft‐pack cells under both internal and external heating conditions [95]. Calorimetry revealed a three‐stage progression: a latent stage (<100°C) with negligible reactivity; an intermediate stage (100–150°C) dominated by SEI and anode exotherms, still reversible with adequate cooling; and a critical stage (>150°C) marked by cathode decomposition and separator melting, culminating in voltage collapse and runaway. Notably, both onset (∼ 100°C) and critical (∼ 150°C) were higher under external heating than internal self‐heating, reflecting the buffering capacity of the cell. A higher SOC reduced these thresholds and intensified the failure severity, whereas localized heating at the current‐collector tabs accelerated hot spot formation and separator shrinkage. Building on these insights, Jin et al. demonstrated that electrode heterogeneity strongly modulated runaway initiation under overcharge conditions [96]. In situ infrared imaging and multi‐physics simulations have shown that regions of reduced porosity or uneven thickness act as hotspots, concentrating current and thermal flux. Such regions promote exothermic side reactions, including electrolyte decomposition and transition‐metal reduction, leading to earlier runaway, peak surface temperatures exceeding 600°C, and more severe collapse compared with uniform cells. Furthermore, heating in the tab region advanced runaway by ∼80 s relative to center‐face heating, and the authors proposed a “thermal sensitivity coefficient” that increased sharply above 80% SOC, offering a potential metric for battery management systems (BMS) safety algorithms.

#### 2.3.2 Internal Heat Accumulation

Unlike thermal abuse driven by direct external heating, internal heat accumulation poses an insidious threat to the safety of LIBs. Even under normal operating conditions, LIBs inevitably generate heat through irreversible electrochemical reactions, ohmic resistance, and entropy changes during cycling [97]. Li et al. provided a comprehensive analysis of the heat generation and transfer processes in LIBs, emphasizing that thermal instability often arises when internal heat generation outpaces the cell's ability to dissipate it [98]. Key contributors to this imbalance include contact resistance at the electrode–collector interface, inhomogeneous current distribution, and impedance growth induced by SEI degradation and lithium plating. In high‐energy‐density formats, particularly large stacked architectures, the intrinsic thermal resistance isolates the cell core from external heat sinks, delaying diffusion and allowing modest heating to elevate core temperatures beyond critical thresholds. Once this occurs, the autocatalytic decomposition of the electrolytes and transition metal oxides can be triggered, setting the stage for thermal runaway. Modeling work by Tulabi and Bubbico revealed that aging exacerbates this imbalance [99]. According to their simulations, aged cells with elevated impedance and reduced ionic mobility exhibit steeper temperature increases, even at nominal C‐rates, together with expanded core–surface gradients that mask the internal heating from surface thermistors. This latency renders conventional thermal monitoring insufficient, allowing incipient runaway to remain undetected until the failure propagates.

Although aging is closely linked to thermal safety, it is difficult to express this relationship using a single universal equation because aging is not a unique state variable. The aging–safety relationship depends on cell chemistry, aging pathway, SOC, state of health (SOH), impedance growth, SEI evolution, lithium plating, gas accumulation, and abuse conditions. Instead, this relationship can be quantitatively evaluated by coupling aging descriptors with thermal‐safety descriptors. Aging descriptors include SOH, capacity retention, internal resistance, charge‐transfer impedance, SEI growth, and lithium plating, whereas thermal‐safety descriptors include thermal runaway onset temperature, onset time, self‐heating rate, heat generation rate, peak temperature, gas evolution, and core–surface temperature gradient.

Recent studies have shown that SOH alone is insufficient to describe aging‐related safety degradation [11]. Cells with similar SOH can exhibit different exothermic onset temperatures and thermal runaway delay times when their aging histories, impedance evolution, or interfacial degradation pathways differ. In addition, decreasing SOH during charging has been reported to reduce the thermal runaway onset temperature from 274.8 ± 9.1°C to 238 ± 1.2°C, shorten the critical time by approximately 1 min, and increase the maximum heat release rate by 37.5–45.5% under high charging conditions [100]. These results indicate that aging‐related safety degradation should be quantified not only by capacity fade, but also by impedance growth, onset‐temperature shift, onset‐time reduction, heat‐release intensification, and thermal‐gradient evolution.

Based on this, recent studies have underscored the decisive role of spatial heat localization and structural vulnerabilities in driving internal instability. Zhou et al. systematically examined how localized heating, when applied at multiple points within a single cell, alters both the threshold and dynamics of thermal runaway onset [93]. Using a dual‐heating‐source protocol, they showed that even with the same total heating power, thermal runaway occurred approximately 28% earlier (2516 s vs. 3514 s) and reached substantially higher peak surface temperatures (∼ 733°C vs 567°C) when heat was concentrated near electrode tabs or separator interfaces (regions characterized by high current density and poor thermal conductivity). Compared with uniformly heated cells, these targeted conditions produced steeper temperature gradients and more aggressive gas venting, highlighting that localized hotspot formation can dictate runaway dynamics independent of the global heating rate. In a complementary study, Chen et al. evaluated LFP cylindrical cells in which the external insulating sleeve near the jelly roll edge was deliberately weakened [87]. Localized current pathways initiate hotspots that rapidly degrade the separator and electrolyte, culminating in casing rupture within 110 s of initiation. The rapid failure propagation demonstrated that the process was governed not only by electrochemical reactions, but also by the coupling of electrical arcing, structural heat confinement, and insufficient edge‐side dissipation. Taken together, these findings reveal that spatially concentrated heating and marginal structural defects can outweigh bulk heat inputs in dictating runaway behavior, underscoring the importance of localized diagnostics and development of targeted thermal‐barrier strategies.

#### 2.3.3 Thermal Propagation

Once thermal runaway is initiated in a single cell, the risk of propagation to adjacent cells becomes a critical safety concern, particularly in high‐energy configurations such as EV battery modules and grid‐scale energy storage systems. Thermal propagation occurs when the intense heat generated by a failing cell induces secondary runaway in neighboring cells, creating a cascading sequence of exothermic reactions that can rapidly compromise the integrity of an entire module or pack [101]. Unlike the initiation of thermal runaway, which is often driven by localized abuse or latent defects, the propagation dynamics are determined by system‐level factors, including inter‐cell spacing, heat transfer pathways, cell geometry, and the effectiveness of thermal isolation strategies. This escalation mechanism has been widely recognized as one of the most destructive failure modes in LIB systems because it transforms a localized incident into a large‐scale thermal and safety hazard [102].

Kong et al. conducted a detailed numerical study to evaluate the propagation behavior of 21700‐format cylindrical LIBs subjected to mild overcharge cycling [103]. Their multi‐physics model captured the coupled thermal and structural evolution of cells from initial abuse to runaway onset and subsequent propagation. Key results showed that reduced inter‐cell spacing or obstructed venting pathways substantially accelerated heat transfer to neighboring cells, shortening the delay time for secondary runaway by more than 50%. Furthermore, the orientation of the vent outlets, whether upward or lateral, strongly influenced both the directionality and severity of the propagation. Complementing these simulations, Ye et al. performed full‐scale experiments on high‐capacity multicell modules under immersion‐cooling conditions [104]. By initiating runaway in a single cell, they compared air‐cooled and dielectric immersion environments. Immersion cooling markedly suppressed the peak temperatures and completely contained the runaway within the trigger cell in several tests. Their multiscale analysis highlighted that the decisive factor was the heat extraction capability at the cell–coolant interface, which disrupted the thermal feedback loop required for sustained propagation.

These studies highlight that thermal propagation is not merely an extension of the initial runaway event but rather a distinct failure mode shaped by the inter‐cell architecture, thermal resistance, and boundary conditions. As LIB systems continue to scale in terms of both capacity and complexity, interrupting the propagation pathways is indispensable for ensuring robust safety performance at the module and pack levels.

## 3 Stages and Mechanism of Thermal Runaway

Thermal runaway in LIBs is a multistage failure process initiated by exothermic decomposition and barrier breakdown, leading to rapid increases in both temperature and internal pressure. Each component, anode, cathode, separator, and electrolyte, contributes to the escalation through heat and gas generation, forming a self‐reinforcing cascade [17]. Understanding the sequence of these reactions is crucial for developing targeted mitigation strategies at both the material and system levels [105, 106].

### 3.1 Overview of Thermal Runaway Mechanisms

**FIGURE 2:**
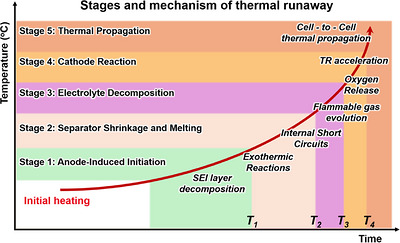
Stages and mechanism of thermal runaway in lithium‐ion batteries. The process evolves from anode‐induced initiation and SEI layer decomposition to separator melting, electrolyte decomposition, and cathode oxygen release, ultimately accelerating into cell‐to‐cell thermal propagation with rapidly rising temperature.

Thermal runaway is a multistage, component‐specific degradation cascade rather than a singular catastrophic event. As depicted in Figure 2, the process often originates from localized thermal or electrochemical instabilities, typically at the anode, and progresses through the sequential decomposition of the SEI, electrolyte vaporization and combustion, separator collapse, and oxygen release from the cathode [107]. With heat accumulation outpacing dissipation, these reactions are amplified through positive feedback, ultimately enabling self‐sustained propagation across adjacent cells [108, 109, 110, 111, 112]. The thermal runaway process in LIBs can be broadly categorized into four temperature‐associated stages.

- **Initiation (T_1_, ∼50–160°C)**: SEI decomposition and reactions between reduced anodes and electrolytes generate low‐level heat and gases.
- **Acceleration (T_2_, ∼120–250°C)**: Separator deformation, electrolyte decomposition, gas generation, and ISCs accelerate heat and pressure buildup.
- **Runaway Reaction (T_3_, ∼180–350°C)**: Strong exothermic reactions involving electrolytes and cathodes dominate, with the contribution of cathode oxygen release varying by cathode chemistry.
- **Propagation (T_4_, after self‐sustained runaway)**: Heat, flame, hot gases, ejecta, and pressure waves transfer to neighboring cells, producing cell‐to‐cell and module‐level cascade failures.

Importantly, the temperature ranges in the T_1_–T_4_ model should be interpreted as overlapping reaction windows rather than fixed thresholds, because each stage is affected by component chemistry, SOC, SOH, electrolyte formulation, and cell format.

The early initiation stage (T_1_) is governed primarily by SEI chemistry and lithiated‐anode reactivity. SEI‐related heat release has been reported to begin in the low‐temperature region of approximately 50–120°C, while carbon or graphite anodes can exhibit heat‐release onset over approximately 80–160°C depending on lithiation state, electrolyte composition, and aging history [108, 109]. In Si‐containing or Li‐metal anodes, early heat and gas generation can be further affected by SEI instability, interfacial area, lithium morphology, and cycling history [110, 111].

The acceleration stage (T_2_) is associated with separator deformation, electrolyte decomposition, gas generation, and ISC formation. Polyolefin separators can undergo shutdown or pore collapse when polyethylene (PE)‐rich domains soften near approximately 130–135°C, while polypropylene (PP)‐rich domains generally melt near approximately 160–165°C [112, 113]. For lithium hexafluorophosphate (LiPF_6_)/carbonate electrolytes, salt‐assisted solvent decomposition and gas‐generating reactions commonly occur across approximately 150–250°C, with more intense evaporation, decomposition, and combustion becoming prominent above approximately 200°C [114, 115, 116, 117]. Ether‐based electrolytes exhibit broader formulation‐dependent behavior, because low‐boiling ethers such as dimethoxyethane (DME) and 1,3‐dioxolane (DOL) can volatilize at relatively low temperatures, whereas concentrated, fluorinated, or localized high‐concentration ether electrolytes can shift decomposition and Li‐metal reactivity to higher temperatures [111].

The runaway reaction stage (T_3_) is dominated by strong exothermic reactions involving electrolytes and cathodes. Ni‐rich layered oxides, represented by LiNi_0.8_Co_0.1_Mn_0.1_O_2_ (NCM811), can release lattice oxygen around approximately 210–250°C, promoting cathode–electrolyte combustion and rapid self‐heating [114, 115, 116, 117]. LFP cathodes exhibit weaker oxygen‐release contribution because the olivine phosphate framework stabilizes oxygen. Reported heat‐release onset temperatures for fully charged LFP cathodes generally fall within approximately 180–250°C, with peak heat release extending over approximately 210–360°C. In LFP/electrolyte mixtures, the initial exothermic temperature can decrease to approximately 225°C, indicating that electrolyte participation can dominate the high‐temperature response [109, 118]. LiMn_2_O_4_ (LMO) cathodes show intermediate behavior between Ni‐rich layered oxides and LFP. Delithiated LMO can exhibit an exothermic peak near approximately 270°C without electrolyte, while LMO/electrolyte systems can show lower onset temperatures of approximately 158–162°C due to electrolyte‐assisted reactions, although their overall heat release is lower than that of layered oxide systems [118].

The propagation stage (T_4_) is defined by hazard transfer to neighboring cells rather than by a single material decomposition temperature. After self‐sustained runaway, heat, flame, hot gases, ejecta, and pressure waves can trigger secondary failure in adjacent cells. Therefore, T_4_ depends strongly on cell format, module spacing, vent direction, thermal barrier design, pack enclosure, SOC, and cooling conditions. This stage‐resolved screening shows that the T_1_–T_4_ model should be used as a component‐ and chemistry‐resolved reaction map, where SEI and anode chemistry control early initiation, separator and electrolyte behavior govern acceleration, cathode chemistry determines the severity of high‐temperature runaway, and module architecture controls propagation.

### 3.2 Anode‐Induced Initiation of Thermal Runaway

The anode–electrolyte interface is frequently the first locus of thermal instability, particularly under high SOC or elevated‐temperature conditions. The two key initiation mechanisms are SEI decomposition and direct redox reactions between the exposed anode surfaces and electrolyte solvents [119, 120]. These reactions are typically localized and exothermic, often evading early detection; however, they play a crucial role in generating heat and gas build‐up that triggers subsequent events, including separator failure and cathode decomposition [121].

#### 3.2.1 SEI Layer Decomposition

The SEI, which forms spontaneously on the anode surface during the initial charge–discharge cycles, is essential for stabilizing LIBs by inhibiting continuous electrolyte reduction [17]. They generally comprise an inner inorganic‐rich layer (Li_2_CO_3_, LiF, and Li_2_O) and an outer organic‐rich region (ROCO_2_Li and ROLi) with a precise composition dictated by the electrolyte formulation and operating conditions. While electrochemically stable under normal cycling, the SEI is known to decompose exothermically at 90–120°C, releasing CO_2_, H_2_, CH_4_, and, in some cases, HF [119]. These gases increase internal pressure and transiently lower interfacial impedance, thereby promoting further reactions. Calorimetric analyses by Liu et al. indicate that SEI decomposition can account for the majority of heat released within this 90–120°C window, particularly during the early stages in graphite anodes [122]. Moreover, SEI breakdown exposes the underlying anode to a fresh electrolyte, initiating parasitic redox reactions that further amplify heat generation [123]. These reactions can be represented by simplified pathways, as follows:

$$Li_{2}CO_{3}\to \;Li_{2}O+CO_{2}\;\mathrm{or}\;\mathrm{CO}↑$$

$$\mathrm{ROC}O_{2}\mathrm{Li}\;\to \;R-H+\mathrm{CO}2↑+\mathrm{Li}2O$$

The impact of SEI decomposition is particularly pronounced in high‐capacity anodes, such as silicon and lithium. These materials undergo substantial volume expansion during cycling, which repeatedly fractures and regenerates the unstable SEI layers. Yoon et al. demonstrated that Si‐rich electrodes exhibit lower decomposition onset temperatures and higher gas evolution rates than graphite analogs under identical heating protocols [110]. In Li metal cells, Xu et al. showed that SEI are even more fragile and thermally unstable owing to inhomogeneous plating and dendrite formation [124]. Thermal‐modeling studies further suggest that SEI breakdown follows a nonlinear, autocatalytic trajectory: once local gas pressure and temperature exceed ∼100 °C, the decomposition rate accelerates sharply, shifting the system from passive heating to active self‐heating. Collectively, these findings indicate that SEI decomposition represents a thermochemical tipping point that transforms the cell from a metastable state into an actively reactive one, thereby initiating a cascade of reactions that define thermal runaway.

#### 3.2.2 Exothermic Reactions between Anode and Electrolyte

Following decomposition of the SEI layer, the freshly exposed anode surface reacts directly with the liquid electrolyte, initiating a cascade of strongly exothermic processes [122]. This step marks the first major thermochemical escalation in thermal runaway, driven by the abundance of reductive species at the anode and the high reactivity of carbonate solvents such as ethylene carbonate (EC), dimethyl carbonate (DMC), and diethyl carbonate (DEC). These reactions typically involve solvent reduction and gas‐phase decomposition, generating CO_2_, CH_4_, and other light hydrocarbons [125, 126].

Using operando differential scanning calorimetry‐Fourier transform infrared spectroscopy (DSC‐FTIR), Lu et al. showed that in NCM/Li‐metal cells, heat release from plated‐Li/electrolyte reactions scales with both the lithium morphology and the electrolyte formulation. High‐surface‐area filamentary lithium was found to drive rapid solvent reduction, producing CO_2_ and C_2_H_4_ as the dominant gaseous products, and releasing tens of joules per gram of additional heat prior to any cathode involvement [127]. Similarly, Song et al. combined accelerating rate calorimetry (ARC) testing with a 3D electrothermal model of 45 Ah NCM811/graphite pouch cells, revealing that negative‐electrode solvent reduction occurs several tens of degrees before cathode decomposition and contributes ∼20% of the total runaway heat [128]. These results highlight that the anode‐electrolyte reactions serve as an ignition stage, dictating the flame‐jetting intensity and pressure surges in the subsequent runaway progression.

### 3.3 Separator Shrinkage and Melting

The separator in LIBs plays a dual role, simultaneously ensuring ionic conductivity and preventing direct anode–cathode contact. Most commercial separators are polyolefin‐based, typically polyethylene (PE) or polypropylene (PP), and are valued for their chemical inertness and high porosity. However, these polymers begin to shrink at ∼120–130°C and melt at 160–170°C, compromising dimensional stability. Such shrinkage erodes the mechanical barrier between the electrodes and precipitates the ISCs [129].

Ren et al. demonstrated via ARC testing of 34 Ah NMC811 pouch cells that separator contraction at ∼135°C coincided with a 25‐fold reduction in cell impedance, inducing an ISC within just 8 s [130]. While shrinkage itself generates little heat, the resulting short‐circuit currents drive intense ohmic heating and localized thermal spikes that quickly accelerate runaway. The severity of this failure mode strongly depends on the separator morphology, thickness, and softening temperature. Kalnaus et al. further identified anisotropic shrinkage of up to 18% in the machine direction at 150°C in commercial PP films, explaining why multilayer separators often tear and localize shorts under mechanical or thermal stress [131]. When the separator collapse coincides with ongoing electrode reactions, the combined heat and gas evolution dramatically amplifies the escalation. To mitigate this risk, ceramic‐coated and hybrid separators, capable of retaining structural integrity with < 5% dimensional change even at 200°C, have been proposed as active thermal‐management solutions.

### 3.4 Electrolyte Decomposition and Combustion

Among various LIB components, electrolytes are arguably the most chemically reactive under thermal stress. Composed of organic carbonate or ether solvents, lithium salts, and a range of functional additives, the electrolyte serves not only as an ionic transport medium but also as a concentrated fuel source for thermal runaway [79]. Once internal temperatures exceed ∼140 °C, electrolyte decomposition accelerates rapidly, producing large volumes of flammable gases and triggering combustion when mixed with oxygen. Unlike the degradation of other cell components, electrolyte reactions are highly exothermic and irreversible, often marking a transition from controlled overheating to catastrophic failure [132, 133]. The electrolyte is intrinsically a multicomponent system, where stability is dictated not by any single constituent, but by the coupled interactions among solvents, salts, and additives. Each component contributes differently to the heat release, gas evolution, and degradation pathways under elevated temperatures. Therefore, a mechanistic understanding of these interactions is critical for the rational design of safer electrolyte formulations [79, 132, 133].

#### 3.4.1 Carbonate‐Based Electrolytes

Carbonate‐based solvents, primarily cyclic and linear carbonates, such as EC, DMC, DEC, and ethyl methyl carbonate (EMC), remain the foundation of commercial LIB electrolytes because of their high dielectric constants and compatibility with graphite anodes. However, their limited thermal stability makes them among the earliest components to undergo decomposition under harsh conditions [79]. Thermal decomposition typically initiates at ∼140–160°C, with the onset strongly dependent on solvent structure and the surrounding cell environment. Fernandes et al., using coupled DSC and micro‐gas shromatography (GC), reported that linear solvents DMC and DEC exhibit exothermic onset near ∼150°C, whereas cyclic EC remains relatively stable until ∼165°C [134]. The evolved gases were CO_2_‑rich (> 60 vol.%) with minor amounts of CO and H_2_, explaining why linear carbonates key drivers of rapid pressure buildup during abuse. On the other hand, Cui et al. showed that a molecular‐anchoring localized high‐concentration electrolyte (3 M lithium bis(fluorosulfonyl)imide (LiFSI) in EC/EMC with a tethered ether additive) postponed the main solvent exotherm to ∼210°C and reduced total vent‐gas volume by ∼45% in adiabatic calorimetry [135]. Infrared and gas analysis confirmed the suppression of phosphorus pentafluoride (PF_5_)‐catalyzed EC ring‑opening, thereby lowering CO_2_ release and halving the self‐heating rate during nail penetration abuse.

#### 3.4.2 Ether‐Based Electrolytes

Ether‐based solvents such as dimethoxyethane (DME), tetraethylene glycol dimethyl ether (TEGDME), and 1,3‐dioxolane (DOL) are central to the development of lithium metal and other next‐generation high‐energy chemistries owing to their low viscosity and good compatibility with lithium metal [136]. However, these benefits come with a critical tradeoff: significantly lower thermal stability [137]. Unlike carbonates, which generally decompose above ∼140°C, ether‐based electrolytes can undergo rapid breakdown between ∼110–130°C [138, 139].

Huang et al. demonstrated that in large‐format Li–S pouch cells, DOL undergoes ring‐opening polymerization at ∼120°C, generating an additional exothermic peak that accelerates self‐heating once decomposition is triggered [138]. These ether‐specific reactions are further intensified by residual moisture or catalytic surfaces such as high‐surface‐area lithium deposits and transition metal particles, both of which amplify heat release and radical generation. Wang et al., using flow‐reactor pyrolysis of DOL (743–1013 K, 150 torr), quantified CH_4_, C_2_H_4_, CO, and CH_2_O as major products and mapped β‐scission and H‐transfer pathways responsible for high gas evolution of ether‐based solvents [140]. Guenther et al. further showed that ether vapors, such as DME and DOL, ignite with oxygen approximately one order of magnitude faster than carbonate vapors under comparable conditions, highlighting their heightened flame propagation risk during overcharge or nail‐penetration abuse [141].

Collectively, ether‐based electrolytes provide electrochemical advantages for advanced systems. However, their inherently low thermal thresholds and rapid vapor‐phase flammability make them critical accelerants of runaway once thermal abuse is initiated.

#### 3.4.3 Salt and Additive Decomposition

While solvents dominate the vapor‐phase fuel generation, lithium salts and electrolyte additives exert strong control over the decomposition pathway and its severity. In particular, the most widely used salt, LiPF_6_, is thermally unstable, even at moderate temperatures [142]. Above ∼60°C, LiPF_6_ dissociates into LiF and PF_5_, the latter acting as a potent Lewis acid that catalyzes solvent breakdown. In the presence of trace moisture, PF_5_ hydrolyzes to form HF, a corrosive species that damages the electrode–electrolyte interface and accelerates structural degradation. In contrast, alternative salts such as lithium bis(fluorosulfonyl)imide (LiFSI) and lithium bis(trifluoromethanesulfonyl)imide (LiTFSI) offer significantly greater thermal and chemical stabilities. Unlike LiPF_6_, these salts lack fluorophosphates and thus do not produce PF_5_ or HF upon decomposition, thereby reducing both the heat release and the formation of corrosive byproducts. Electrolytes based on LiFSI or LiTFSI consistently exhibited slower exothermic rates and fewer acidic degradation products, making them suitable for high‐temperature or flame‐retardant applications [143].

Electrolyte additives further modulate the thermal behavior, often with complex or even dual effects. For example, Lee et al. reported that vinylene carbonate (VC) undergoes radical polymerization near ∼140°C, releasing CO_2_ and adding an exothermic peak to DSC traces [144]. Kim et al. found that fluoroethylene carbonate (FEC), which is beneficial for SEI formation, undergoes defluorination in LiPF_6_ electrolytes at elevated temperatures, generating HF and accelerating transition metal dissolution, ultimately worsening the severity of thermal runaway [145].

### 3.5 Cathode Decomposition and Oxygen Evolution

Cathode decomposition is one of the most hazardous events in LIB failure, as phase transitions at elevated temperatures are accompanied by lattice oxygen release, which fuels combustion and accelerates the breakdown of adjacent components [146]. The severity of this process strongly depends on cathode chemistry.

Lithium cobalt oxide (LiCoO_2_, LCO), one of the earliest and most widely studied commercial cathodes, remains prevalent in portable electronics owing to its stable voltage profile, reliable cycling, and predictable electrochemical behavior [147]. Furushima et al. showed that chemically de‐lithiated Li_0.65_CoO_2_ begins releasing lattice oxygen slightly above 250°C, with DSC exotherms at ∼190°C and ∼290°C corresponding to layered‐to‐spinel and spinel‐to‐rock‐salt transitions, respectively [148]. These transformations collapse the Li layers and liberate O, which promptly oxidizes the neighboring electrolyte. Jiang and Dahn reported ARC measurements of LCO electrodes charged to 4.2 V in EC/DEC electrolyte, observing self‐sustained exothermic activity as low as ∼150°C, attributed to rapid reactions between freshly released oxygen and carbonate solvents, which sharply elevate internal pressure [149]. Sun et al. further demonstrated that trace Co dissolution catalyzes ethylene carbonate oxidation, shifting its decomposition onset downward by ∼15°C and reinforcing a feedback loop in which cathode degradation and electrolyte breakdown mutually intensify heat release [150].

Ni‐rich layered oxides (NCM and nickel cobalt aluminuum oxide (NCA) with ≥ 80% Ni in composition) exhibit the highest thermal reactivity among layered cathodes and are key drivers of thermal runaway propagation. Wu et al. observed that overcharged LiNi_0.8_Co_0.15_Al_0.05_O_2_ rapidly transforms from a layered structure to spinel and then rock‐salt above ∼ 200°C, releasing lattice oxygen and producing a high‐enthalpy exotherm [151]. Similarly, Oh et al. reported ARC measurements on pouch cells with ≥ 90% Ni cathodes, showing oxygen evolution initiating at ∼180°C and a twofold increase in peak heat‐release rate when SOC increased from 60% to 100%, confirming that oxygen–electrolyte reactions dominated once lattice collapse began [152]. However, lower‐Ni NCMs are known to follow the same pathway at higher temperatures, underscoring the Ni content dependence [151].

In contrast to layered cathodes, spinel LiMn_2_O_4_ (LMO) and LFP exhibit greater thermal stability, which is characterized by lower heat output and delayed oxygen evolution under equivalent conditions [109, 118, 153]. Spinel LMO, which balances moderate energy density with improved safety, remains common in power tools and cost‐sensitive EVs [154, 155]. Wang et al. showed that delithiated LMO releases oxygen just above ∼210°C through Mn^3+^ → Mn^2+^ + Mn^4+^ disproportionation, producing a modest DSC exotherm and soluble Mn^2^^+^ that can migrate into the electrolyte [154]. Röder et al. reported 2 Ah pouch cells composed of a Li[Ni_0.33_Co_0.33_Mn_0.33_]O_2_/LiMn_2_O_4_ based cathode using ARC and DSC. They reported that the dominant exothermic behavior was driven by reactions between the delithiated cathode and the electrolyte, with major heat release occurring at temperatures approaching 215°C. The vented gases included CO_2_ among other species, and no external flame was observed during the thermal event. The maximum self‐heating rate of the cell was notably lower than that typically observed in systems based on Ni‐rich layered oxide cathodes [155].

LFP is widely regarded as the safest commercial cathode due to its robust olivine lattice, which resists oxygen release even under full delithiation and heating to ∼300°C [109]. Bugryniec et al. found that LFP cathodes generate heat between ∼180–250°C, peaking at 210–360°C with a total release of 150–340 J g^−1^, while full 18650 cells evolved only ∼0.5 g of oxygen during runaway compared with ∼3.25 g for LCO. This difference arises from the strong P─O bonds that suppress oxygen release from the olivine framework. Sun et al. further dissected the thermal runaway sequence of LFP cells at different SOC levels via ARC: (1) Before onset of abnormal heat generation (T_1_), cell temperature rises due to external heating, with no significant internal reactions and stable voltage. (2) After T_1_, the SEI decomposition initiates self‐heating and side reactions, accompanied by a slight voltage drop and fluctuation. (3) Thermal runaway begins, gas venting occurs, the reactions are temporarily slow, and the voltage continues to fluctuate. (4) Above 261°C, high‐SOC cells exhibited a rapid temperature surge, whereas 50% SOC cells bypassed this stage. (5) Beyond 302°C, internal shorting drove voltage to 0 V, releasing intense heat and reaching peak temperature [156].

### 3.6 Self‐Heating and Thermal Propagation

The final stage of thermal runaway is characterized not only by localized chemical decomposition but also by the onset of self‐sustaining heat generation and inter‐cell propagation. Once the exothermic reactions accumulate beyond a critical threshold, the heat produced outpaces heat dissipation, initiating a positive feedback loop. This self‐heating behavior accelerates the decomposition kinetics and, in large‐format cells or battery packs, can trigger cascading failures across neighboring units [157, 158]. Unlike earlier component‐level degradation, this stage reflects the dynamic coupling between thermal, chemical, and physical processes evolving over time [106, 159]. The severity of this regime is determined by the rate of heat generation, thermal mass, and insulation of the system, and the spatial distribution of the reactive sites [18].

#### 3.6.1 Self‐Heating and Runaway Acceleration

Self‐heating represents the tipping point at which the internally generated heat surpasses the external dissipation, turning the cell into its own heat source. ARC typically defines the onset when the self‐heating rate (dT/dt) exceeds ∼0.02°C min^−1^, while full runaway is conventionally classified once dT/dt surpasses 10°C min^−1^ [160]. Localized hotspots arising from contact resistance, electrode heterogeneity, or micro‐cracks serve as ignition sites. Operando micro‐Raman imaging has shown that such hotspots can trigger internal shorts long before the bulk temperature reaches global onset [161].

Following the onset of self‐heating, autocatalytic reactions, including electrolyte oxidation, anode–electrolyte redox reactions, and cathode oxygen release, accelerated sharply. For example, ARC measurements on a 25 Ah NMC pouch cell revealed a transition in self‐heating rate from < 2°C min^−1^ to >30°C min^−1^ once the temperature entered the 200–220°C range [162]. In high‐capacity 26650 cells, the rate can exceed 100°C min^−1^ within the 240–280°C window, with complete runaway typically occurring in under one minute under adiabatic conditions [163]. Collectively, these findings confirm that self‐heating constitutes a decisive, nonlinear tipping point that separates benign overheating from violent, irreversible runaway behavior.

#### 3.6.2 Cell‐to‐Cell Thermal Propagation

While self‐heating defines the failure of a single cell, propagation dictates whether the event remains localized or evolves into a system‐level catastrophe [157]. Once runaway is triggered in an 18650 cell, heat transfer through metallic casings and tabulated networks becomes dominant. Experiments indicate that maintaining cylindrical cells at least 4 mm apart (or 6 mm at 100% SOC) prevents vertical propagation initiated by a bottom heater [164]. Side‐heating tests using 200–400 W cartridge heaters demonstrated that approximately 10–14 kJ of transferred energy (roughly 200 W sustained for 300–600 s) was required to induce runaway in an adjacent cell with 2 mm spacing [165]. Increasing the separation to 5 mm approximately doubled the energy threshold and delayed the venting by more than 150 s.

Even under tight spacing, cascading failure can be avoided if the peak temperature of neighboring cells remains below ∼ 200°C [157]. López et al. observed that no secondary 18 650 cells breached this threshold despite local heat fluxes approaching 60 W when the trigger cell failed. Beyond the conductive transfer, the jet vent gases provide a secondary propagation pathway [166]. Mishra et al. have shown that plumes of vented gases significantly accelerate propagation in enclosed modules, especially when venting is constrained, highlighting the coupled roles of gas ejection, spacing, and system architecture in determining cell‐to‐cell propagation [167].

In large‐format battery systems such as EV packs and stationary ESSs, the transition from single‐cell failure to multi‐cell propagation marks a pivotal escalation point, converting localized degradation into a full‐scale thermal catastrophe. This propagation is governed not only by heat transfer but also by complex, coupled interactions involving thermal gradients, mechanical constraints, gas‐phase jetting, and electrochemical feedback between neighboring cells. As such, unraveling the multi‐physics coupling that mediates cell‐to‐cell propagation, particularly under varying states of charge, spatial configurations, and boundary conditions, is essential for devising both predictive diagnostics and robust containment architectures. A comprehensive understanding of these propagation pathways is indispensable for transitioning from reactive to preventive safety paradigms in next‐generation battery systems.

Collectively, the mechanisms of thermal runaway reveal a tightly coupled cascade that evolves from localized instabilities at the anode–electrolyte interface to full‐scale, system‐level failures driven by heat accumulation, gas evolution, and structural collapse. While each component exhibits distinct failure kinetics, their interactions converge into a nonlinear feedback network that ultimately dictates the severity of runaway. Recognizing thermal runaway as a systemic rather than component‐level phenomenon underscores the urgent need for integrated mitigation approaches. Accordingly, the following sections of this review focus on material, structural, and system‐level engineering strategies aimed at interrupting this cascade and embedding intrinsic safety into the design of next‐generation LIBs.

## 4 Materials for Safety Enhancement

### 4.1 Ni‐Rich Layered Oxide Cathodes

As mentioned in the previous section, LCO, NCM, LMO, and LFP are the cathodes frequently used and studied in the LIB area currently. However, depending on the operating environment and conditions of charge/discharge, these materials can undergo phase transition and lattice oxygen releasing problems [153, 168, 169, 170], which lead to serious side reactions with electrolytes and transition‐metal reduction [171]. Moreover, those cause lattice collapse and transition‐metal dissolution at the cathode surface. These phenomena not only deteriorate the electrochemical performance of the battery but also raise the battery temperature and accelerate thermal runaway, leading to LIB failure at the end [172, 173]. To increase the bulk and surface stability of the cathode against the thermal, various strategies such as coating, doping, and modifying the synthesis process of the cathode have been proposed.

#### 4.1.1 Strategy I: Coating

**FIGURE 3:**
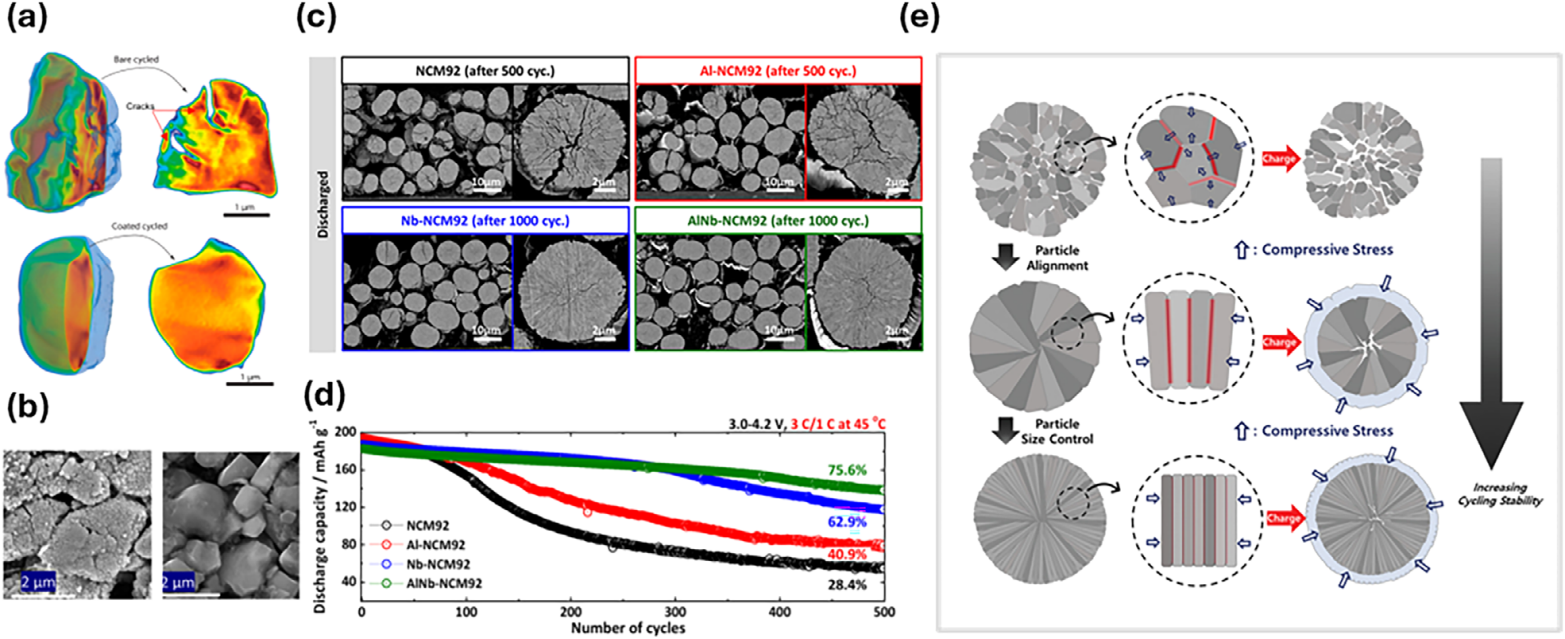
(a) Intragranular microcracks and surface reconstruction analysis. Ptychographic X‐ray computed tomography of material particles after 200 cycles between 2.8 and 4.6 V for the uncoated and coated single‐crystalline (SC)‐NMC83 (Adapted from Liu et al., ACS Nano 16 (2022): 14527–14538). (b) SEM images of the post cycled materials: uncoated and coated SC‐NMC83 (Adapted from Liu et al., ACS Nano 16 (2022): 14527–14538). (c) Cross sectional SEM images of discharged NCM92, Al‐NCM92, Nb‐NCM92, and AlNb‐NCM92 cathode particles (Adapted from Lee et al., ACS Energy Lett. 9 (2024): 740–747). (d) Cycling performance of pouch‐type full‐cells featuring graphite anodes 3 C charged, 1 C discharged rate at 45°C (Adapted from Lee et al., ACS Energy Lett. 9 (2024): 740–747). (e) Schematic of secondary particles consist of (top) randomly oriented, (middle) radially oriented, and (bottom) size‐refined primary particles (Adapted from Ju et al., ACS Energy Lett. 8 (2023): 3800–3810).

Surface coatings with thermally and mechanically stable materials have been widely explored to mitigate the cathode degradation arising from side reactions at the cathode–electrolyte interface. Ceramic and polymeric materials have also been investigated as potential protective layers [174, 175, 176, 177]. Wang et al. proposed perovskite oxides such as La_4_NiLiO_8_ as promising coatings for cathodes owing to their structural flexibility and superior Li^+^ conductivity [178]. A La_4_NiLiO_8_ coating effectively accommodates the crystal mismatch and suppresses intergranular cracking during the H2–H3 transition, even under a 4.5 V cutoff, where anisotropic volume change and c‐axis contraction typically induce severe mechanical damage. Such coatings also raise the decomposition temperature and reduce gas evolution, thereby enhancing the thermal stability. Polymeric coatings can also offer complementary benefits: amorphous poly(3,4‐ethylenedioxythiophene) (PEDOT), for example, improves particle integrity and cycling stability at 4.6 V and 45°C by restraining intragranular cracking and suppressing surface corrosion (Figure 3a,b) [179]. Although coatings are effective in enhancing high‐temperature operation, the deterioration of the coating layer itself and its vulnerability to external damage remain challenges, underscoring the need for complementary stabilization strategies.

#### 4.1.2 Strategy II: Doping

Bulk doping with foreign elements is another effective route for enhancing the thermal and electrochemical stabilities of Ni‐rich cathodes. Redox‐inactive and highly electronegative cations such as Mg^2+^, Ti^4+^, Zr^4+^, and Nb^5+^ have been incorporated into the transition metal (TM) layer to lower the Ni content and suppress instability [180, 181, 182, 183]. Lv et al. reported that even trace Mg substitution (<0.01 mol) can suppress particle cracking and TM dissolution under 4.5 V operation [184], while Nb doping near the surface forms a passivation layer that stabilizes operation at 50°C [185]. The introduction of electronegative dopants at oxygen sites has also been reported to mitigate TM migration by strengthening TM–X (X = dopant) bonds [183]. Yang et al. showed the anisotropic alleviation in the lattice strain accumulation and irreversible lattice oxygen release by fluorinating the cathode surface and creating the F‐aggregated interface. Substituting F for O anchors the internal transition‐metal ions through the strong bond between the transition‐metal and F and creates a thin and stable cathode electrolyte interface (CEI) by the fluorinated layer. Therefore, in an operating condition of 2.7–4.5 V, cation mixing at the particle surface, microcrack propagation, CO_2_ and heat dissipation are significantly suppressed.

In addition to compositional tuning, doping influences particle morphology. Smaller and more uniform primary particles are favored to suppress microcrack propagation, a feature that can be achieved using high‐valent dopants such as Nb^5+^, Ta^5+^, or Mo^6+^, which inhibit grain coarsening [186]. For example, Lee et al. showed that co‐doping with Nb^5+^ and Al^3+^, combined with optimized calcination, produced radially oriented rod‐shaped primary particles. Al^3^^+^ dispersed homogeneously, whereas Nb^5^^+^ segregated to grain boundaries, collectively reducing particle size and enhancing structural robustness and cycling durability (Figure 3c,d). Integrating coating and doping approaches enables the simultaneous stabilization of both the bulk and surface regions [186, 187, 188].

#### 4.1.3 Strategy III: Synthetic Control

Tailoring synthetic protocols represents another strategy for enhancing the safety of Ni‐rich cathodes. Controlling the particle morphology can mitigate the anisotropic lattice strain accumulated during cycling [189]. For example, applying controlled air pressure during calcination yielded denser spherical secondary particles composed of smaller and more uniform primary grains, as shown in Figure 3e [190]. Such structures alleviate local strain buildup, suppress TM dissolution, and preserve particle integrity even at 55°C. Moreover, the mechanical stability of the cathode was enhanced by the formation of long rod‐shaped particles through internal stress distribution during charge/discharge [191]. Ryu et al. demonstrated that the rod‐shaped principal particles can be more tightly packed along the radial direction into spherical secondary particles. Attributed to the rod‐shaped particles, the cathode not only facilitates Li migration but also has higher particle integrity. Furthermore, this suppresses internal strain due to the H2‐H3 phase transition and prevents the formation of microcracks and electrolyte penetration into the particles.

Gradient composition engineering offers several advantages. A gradual Ni concentration gradient within the cathode particles can mitigate capacity fading and thermal instability [192]. This is typically achieved using different TM precursors during co‐precipitation, resulting in core–shell structures. Compared to conventional NCM811 with a uniform Ni valence distribution, gradient‐structured NCM811 positions low‐valent Ni within the bulk rather than at the surface, thereby improving the cycle stability and thermal tolerance. Prolonged calcination facilitated Mn─O bond strengthening and oxygen diffusion to the core, maintaining Ni^2+^ enrichment at the surface. Beyond Ni valence gradients, tailoring the radial distribution of Co and Mn has also proven beneficial: Co‐enriched surfaces and Mn‐enriched cores suppress microcrack formation and enhance the overall structural integrity [193].

The cathode‐side strategies discussed in this section improve thermal safety by mitigating different stages of cathode degradation. Surface coatings mainly suppress cathode–electrolyte reactions and stabilize the particle surface, whereas bulk doping and compositional modification reinforce the crystal lattice and delay oxygen release. Morphology control, concentration‐gradient structures, and single‐crystal designs further reduce microcrack formation and limit the exposure of fresh reactive surfaces during cycling. Because these approaches differ in thermal stability, cycling retention, processing complexity, and scalability, their practical relevance cannot be assessed using a single metric. Table 2 therefore compares representative cathode modification strategies in terms of their safety mechanisms, performance indicators, scalability, and key limitations.

**TABLE 2:** Comparative summary of cathode modification strategies for thermal safety enhancement.

Strategy	Representative approach	Main safety mechanism	Representative safety or performance indicator	Cost/scalability	Key limitation
Surface coating	Ceramic, oxide, phosphate, polymeric, or conductive coating layers on Ni‐rich cathodes	Suppresses cathode–electrolyte side reactions, surface reconstruction, transition‐metal dissolution, and gas evolution	Increased decomposition temperature, reduced gas evolution, suppressed microcracking, improved high‐voltage cycling stability	Moderate to high, depending on coating method and precursor cost	Incomplete coverage, coating fracture, interfacial resistance, degradation during long‐term cycling
Bulk doping	Mg, Ti, Zr, Nb, Al, B, F, or other dopants in the transition‐metal or oxygen lattice	Reinforces lattice stability, suppresses phase transition, delays oxygen release, and reduces cation mixing	Higher thermal tolerance, reduced H2–H3 transition strain, improved capacity retention at high voltage or elevated temperature	Moderate, compatible with precursor‐level synthesis but composition control is required	Excessive doping can reduce capacity, increase impedance, or complicate composition optimization
Dual or multi‐element doping	Simultaneous introduction of complementary dopants such as Al/Nb or other synergistic combinations	Combines surface stabilization and bulk lattice reinforcement	Reduced crack formation, improved particle integrity, enhanced high‐temperature cycling	Moderate, but process control is more complex than single‐element doping	Requires precise dopant distribution and may increase synthesis cost
Morphology and primary‐particle engineering	Single‐crystal cathodes, radially oriented particles, refined primary particles	Reduces intergranular cracking, limits fresh surface exposure, and improves mechanical robustness	Lower microcrack density, improved cycling at high voltage, suppressed electrolyte penetration	Moderate to limited, depending on synthesis reproducibility and particle‐size control	Lower rate capability in large particles, slower Li diffusion, difficulty in scalable morphology control
Concentration‐gradient or core–shell design	Ni‐rich core with Mn/Co‐enriched or lower‐Ni surface regions	Stabilizes surface chemistry while retaining high bulk capacity	Improved thermal tolerance, reduced surface reactivity, suppressed transition‐metal dissolution	Moderate, based on co‐precipitation but requires gradient control	Complex precursor synthesis, possible gradient fading, trade‐off between capacity and stability

### 4.2 Anode

#### 4.2.1 Graphite

Carbon‐based anodes, particularly graphite, remain the most widely used anodes in LIBs owing to their low cost, high electrical conductivity, and reliable Li storage capability [194, 195]. Compared with Si‐based or Li‐metal anodes, graphite exhibits higher thermal stability; however, its safety can still be compromised by SEI decomposition under elevated‐temperature, high‐voltage, or repeated‐cycling conditions [110, 195, 196, 197].

**FIGURE 4:**
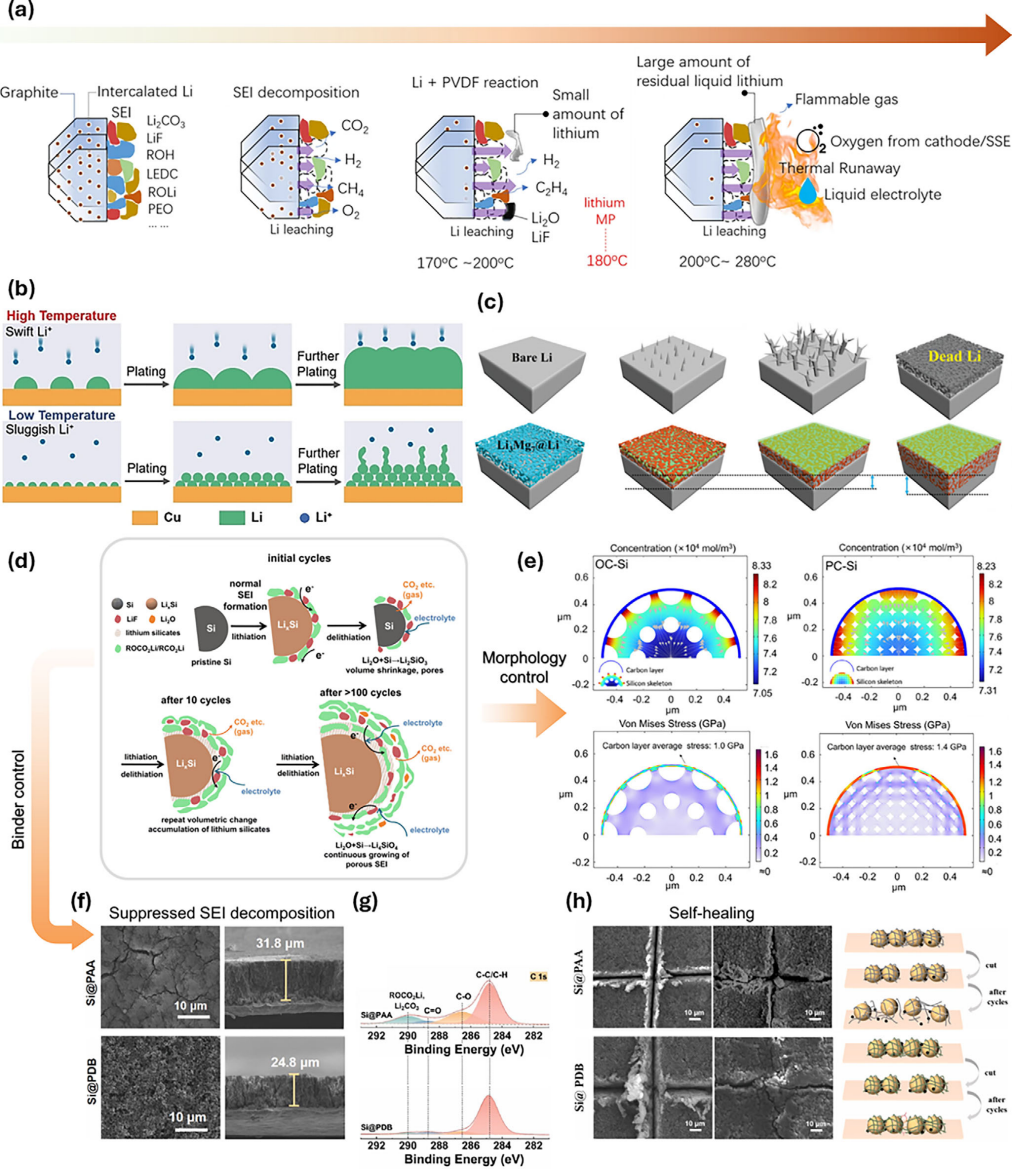
(a) Thermal evolution of lithiated graphite anodes revealed by multiple in situ characterizations (Adapted from Liu et al., Nat. Commun. 12 (2021): 4235). (b) Schematic illustration of the proposed Li nucleation and growth mechanism (Adapted from Yan et al., Angew. Chemie Int. Ed. 58 (2019): 11364–11368). (c) Li deposition behavior on bare Li and Li_3_Mg_7_‐coated Li anode (Adapted from Zhang et al., Adv. Funct. Mater. 31 (2021): 2009712). (d) SEI growth on Si anode particles and its desirable interface (Adapted from Fang et al., Energy Environ. Sci., 17 (2024): 6368–6376). (e) Simulated Li^+^ distribution and Von Mises stress of the origami capsule Si and polynuclear capsule Si electrodes (Adapted from Li et al., Adv. Mater. 37 (2025): 2503745). (f) The top view of surface and cross section SEM images of Si@PAA and Si@PDB electrodes and (g) high resolution X‐ray photoelectron spectroscopy (XPS) spectra of C 1s in each electrodes after 50 cycles. (h) The top‐viewed SEM images of Si@PAA and Si@PDB before and after the scratch with scheme of self‐healing mechanism (Adapted from Geng et al., Journal of Power Sources 630 (2025): 236128).

The SEI originates from electrolyte decomposition during the initial cycle and plays a protective role by suppressing continuous electrolyte reduction, making it essential for long‐term interfacial stability [198, 199, 200]. However, the SEI is not static. It can decompose, facture and reform during repeated cycling or thermal exposure, generating exothermic reactions with the electrolyte (Figure 4a). SEI cracks allow electrolyte penetration and direct contact with lithiated graphite, which can promote solvent co‐intercalation, graphite exfoliation, and further interfacial degradation [122, 198]. These parasitic reactions accelerate heat generation and increase the risk of thermal runaway [201, 202]. Therefore, graphite‐related safety strategies should focus on forming a robust SEI and maintaining interfacial integrity to suppress exothermic anode–electrolyte reactions.

#### 4.2.2 Lithium Metal

Lithium metal has re‐emerged as a highly attractive anode owing to its ultrahigh theoretical capacity of ∼3860 mAh g^−1^ and the lowest known redox potential of −3.04 V vs. standard hydrogen electrode (SHE) [203, 204, 205]. However, uncontrolled dendritic Li growth remains a critical safety challenge. Li nucleates unevenly on the metal surface and preferentially grows at protrusions or locally intensified current‐density regions, eventually penetrating the separator and causing ISCs and catastrophic thermal runaway (Figure 4b) [204]. Therefore, suppressing nonuniform Li nucleation and dendrite formation during repeated plating/stripping is central to mitigating ISC‐induced thermal instability in Li‐metal anodes.

#### 4.2.3 Si‐Based Composites

Si‐based anodes offer another pathway for high‐energy‐density LIBs. In crystalline/amorphous composite structures, amorphous Si can accommodate up to 4.4 Li per host atom, corresponding to a theoretical capacity of approximately 4200 mAh g^−1^ [206]. However, lithiated Si undergoes extreme volume expansion, reaching up to ∼400% during cycling [207, 208]. Repeated expansion and contraction generate mechanical stress, causing particle cracking, pulverization, and continuous SEI rupture and regeneration [209, 210, 211]. The resulting increase in interfacial surface area accelerates electrolyte side reactions, thickens the SEI, impedes Li^+^ transport, and promotes gas‐generating parasitic reactions [212, 213]. From a thermal safety perspective, repeated SEI rupture continuously exposes fresh reactive Li_x_Si surfaces to the electrolyte, thereby increasing heat generation, gas evolution, and the probability of early thermal runaway initiation.

Collectively, graphite, Li‐metal, and Si‐based anodes exhibit distinct thermal runaway‐related failure triggers. Graphite is mainly associated with SEI decomposition and exothermic anode–electrolyte reactions, Li metal with dendrite‐induced ISCs, and Si‐based anodes with volume‐expansion‐induced SEI rupture and regeneration. Accordingly, the following anode‐side safety strategies are reorganized into three corresponding categories: SEI stabilization, Li dendrite suppression, and volume‐expansion suppression.

#### 4.2.4 Strategy I: SEI Stabilization

As discussed in Section 3.2, SEI decomposition on the anode surface is a critical trigger for the early stage of thermal runaway. Although SEI stability is relevant to most anode chemistries, its safety role is particularly direct for graphite anodes, where SEI breakdown exposes lithiated graphite to the electrolyte and initiates exothermic interfacial reactions. Therefore, this section focuses on graphite‐centered SEI stabilization as Strategy I, aiming to form an interphase that is chemically robust, mechanically durable, and resistant to solvent co‐intercalation and thermal decomposition [120, 122].

For graphite anodes, SEI stabilization should not be viewed simply as a method for improving cycling stability. A stable SEI also functions as a thermal safety layer by reducing direct contact between lithiated graphite and the electrolyte, suppressing solvent co‐intercalation, and delaying heat‐generating interfacial reactions. Artificial protective interphases are therefore useful because they regulate the graphite/electrolyte contact before uncontrolled electrolyte decomposition occurs. Feng et al. applied an Al_2_O_3_ coating layer as a preformed SEI on natural graphite, demonstrating that inorganic surface modification can improve interfacial stability and reduce irreversible side reactions [214].

More direct evidence for thermal‐safety‐oriented SEI stabilization was demonstrated by Wei et al., who introduced a Mo_2_N–MoP nanolayer on amorphous carbon‐coated graphite to resolve the trade‐off between fast‐charging capability and high‐temperature stability [215]. In this design, the Mo_2_N phase provides a uniform surface potential for Li‐ion transport, while polar Mo─N and Mo─P bonds in MoP reduce the desolvation energy barrier and suppress solvent co‐intercalation and SEI fracture. The modified interface also promotes the formation of a thermally robust LiF‐rich SEI. DSC measurements showed that amorphous carbon‐coated graphite exhibit∖ed a sharp exothermic peak at 247.0°C with a heat release of 74.4 J g^−1^, whereas Mo_2_N–MoP‐modified graphite showed markedly reduced exothermic heat releases of 26.33 and 19.36 J g^−1^. These results indicate that artificial interphase engineering can reduce exothermic reactivity at the graphite/electrolyte interface and improve the thermal safety margin of graphite anodes.

Taken together, SEI layer stabilization addresses the graphite‐related failure pathway by delaying SEI decomposition, reducing anode–electrolyte heat release, suppressing solvent co‐intercalation, and maintaining interfacial integrity during cycling and thermal stress. Therefore, graphite SEI stabilization should be evaluated not only by electrochemical metrics such as capacity retention and rate capability, but also by safety‐related descriptors such as exothermic onset behavior, heat release, gas generation, and resistance to repeated SEI fracture.

#### 4.2.5 Strategy II: Li Dendrite Suppression

In Li‐metal anodes, the dominant safety risk is governed by the morphology of Li deposition. During repeated plating and stripping, spatially nonuniform Li‐ion flux and local overpotential induce preferential Li nucleation at protrusions or high‐current‐density regions. These protrusions further intensify the local electric field, accelerating dendritic Li growth and increasing the probability of separator penetration, ISCs, and rapid thermal runaway [204]. Therefore, Li‐metal safety strategies should focus on homogenizing Li‐ion flux, reducing nucleation‐barrier heterogeneity, and stabilizing Li plating/stripping before dendrites evolve into internal short‐circuit pathways.

Interfacial modification provides an effective route to regulate Li nucleation at the metal surface. Zhang et al. introduced a lithophilic Li_3_Mg_7_ interlayer to regulate the Li‐metal interface and guide uniform Li deposition (Figure 4c) [216]. During repeated cycling, Mg atoms diffuse into the Li metal and form a lithophilic Li_x_Mg_y_ alloy, which lowers the Li nucleation barrier and promotes spatially uniform Li plating. This interfacial regulation suppresses parasitic reactions and dendritic growth while maintaining the morphological stability of the Li‐metal anode during plating and stripping.

Current‐collector modification can also control Li deposition by regulating the initial nucleation environment. Ding et al. applied nanoporous zeolitic imidazolate framework‐62 (ZIF‐62) glass to a Cu current collector [217]. The isotropic and grain‐boundary‐free structure of the ZIF‐62 glass promotes homogeneous Li nucleation and compact Li deposition without noticeable dendrite formation. By reducing localized Li accumulation and preventing preferential dendrite growth, such current‐collector engineering lowers the probability of separator puncture and dendrite‐induced ISCs.

Accordingly, Li dendrite suppression addresses the Li‐metal‐specific failure pathway by controlling where and how Li is deposited, rather than merely blocking dendrites after they form. Effective Li‐metal safety design should therefore be evaluated using not only Coulombic efficiency and cycling stability, but also safety‐relevant descriptors such as nucleation overpotential distribution, Li deposition uniformity, inactive Li accumulation, dendrite penetration tendency, and ISC onset under abuse conditions.

#### 4.2.6 Strategy III: Volume‐Expansion Suppression

In Si‐based anodes, the dominant safety issue is severe volume expansion during lithiation. Repeated expansion and contraction generate interparticle stress, particle cracking, pulverization, and continuous SEI rupture. The regenerated SEI repeatedly exposes fresh Li_x_Si surfaces to the electrolyte, supplying reactive interfaces for exothermic side reactions, gas evolution, and heat accumulation (Figure 4d) [110, 209, 210, 211]. Therefore, suppressing volume expansion is essential not only for improving cycle life, but also for reducing the anode‐driven thermal runaway initiation.

Conventional approaches such as Si nanosizing, SiO_x_ formation, and Si/C composite design have been widely explored to mitigate mechanical degradation [218, 219, 220, 221]. However, these approaches involve safety‐relevant trade‐offs. Nanosizing can reduce absolute strain at the particle level, but the increased specific surface area promotes parasitic reactions with the electrolyte and repeated SEI growth. SiOx and Si/C composites can improve structural stability, but they may still suffer from irreversible byproduct formation, low initial Coulombic efficiency, coating failure, or incomplete suppression of interfacial degradation. Therefore, recent Si‐anode safety strategies have increasingly focused on particle‐level structural elasticity and electrode‐level mechanical cohesion [222, 223].

Particle‐level structural design can mitigate volume‐expansion‐induced interfacial instability by distributing mechanical stress more uniformly within the Si architecture. Li et al. proposed an origami capsule Si architecture to improve structural flexibility and connectivity of Si anodes (Figure 4e) [222]. The built‐in nanopores and capsule morphology enabled more uniform stress distribution and maintained conductive connectivity during lithiation and delithiation. Compared with conventional secondary Si structures, the origami capsule architecture reduced local stress concentration and suppressed repeated SEI regeneration. The OC‐Si anode showed a low swelling ratio of approximately 14.7% during the first full lithiation, demonstrating that geometrically designed Si architectures can reduce volume‐expansion‐induced safety risks.

Binder engineering provides a complementary electrode‐level strategy by maintaining interparticle cohesion and suppressing electrode delamination during repeated cycling. Even when Si particles are structurally optimized, weak binder–particle adhesion can allow contact loss, crack propagation, and repeated exposure of fresh Si surfaces to the electrolyte. Geng et al. introduced dopamine and boric acid into the poly(acrylic acid) (PAA) backbone to construct a three‐dimensional dynamic hydrogen‐bonding network, yielding the PDB binder (Figure 4f–h) [223]. The PDB binder improved adhesion between Si particles and the electrode matrix, suppressed the particle expansion–crack propagation–surface area increase sequence, and reduced SEI regeneration after cycling. The self‐healing behavior of the binder further helped recover mechanical integrity after damage, thereby decreasing the formation of newly exposed reactive interfaces.

From a safety‐design perspective, volume‐expansion suppression addresses the Si‐specific failure pathway by limiting mechanical fracture, repeated SEI reconstruction, and exothermic anode–electrolyte reactions. Si‐anode safety should therefore be evaluated not only by capacity retention and swelling ratio, but also by particle fracture resistance, electrode‐level adhesion, SEI regeneration rate, gas evolution, and heat release from freshly exposed Li_x_Si surfaces.

The anode‐side strategies discussed in this section target different failure triggers depending on the anode chemistry. In graphite anodes, thermal safety is primarily governed by SEI stability and exothermic reactions between lithiated graphite and the electrolyte. In Li‐metal anodes, the dominant concern is dendrite‐induced separator penetration and internal short‐circuit formation. In Si‐based anodes, severe volume expansion causes particle fracture, repeated SEI regeneration, gas evolution, and heat accumulation. Therefore, anode safety strategies should be compared according to the specific failure pathway they suppress, rather than treated as interchangeable interfacial modifications. Table 3 summarizes the correspondence among anode chemistry, dominant safety trigger, mitigation strategy, and remaining practical challenges.

**TABLE 3:** Comparative summary of anode‐related safety strategies.

Anode material	Dominant safety trigger	Corresponding strategy	Representative safety descriptor	Scalability	Key limitation
Graphite	SEI decomposition, solvent co‐intercalation, exothermic reactions between lithiated graphite and electrolyte	SEI layer stabilization through surface coating, artificial interphase construction, or surface chemistry regulation	Exothermic onset behavior, heat release, SEI fracture resistance, gas generation, solvent co‐intercalation suppression	High to moderate, depending on coating chemistry and process compatibility	Coating uniformity, interfacial resistance, long‐term durability under fast charging or elevated temperature
Li metal	Nonuniform Li deposition, dendrite growth, separator penetration, ISC formation	Li‐ion flux regulation and dendrite suppression through interfacial or current‐collector modification	Nucleation overpotential distribution, Li deposition uniformity, inactive Li accumulation, dendrite penetration tendency, ISC onset	Moderate to limited	High Li reactivity, excess Li requirement, pressure sensitivity, electrolyte dependence, manufacturability
Si‐based anode	Volume expansion, particle cracking, pulverization, repeated SEI rupture and regeneration	Volume‐expansion suppression through particle architecture and binder engineering	Swelling ratio, particle fracture resistance, electrode adhesion, SEI regeneration rate, gas evolution, heat release from exposed Li_x_Si surfaces	Moderate, depending on material design and binder compatibility	Low initial Coulombic efficiency, electrode swelling, process complexity, maintaining high loading and electrode integrity

### 4.3 Electrolyte

Conventional linear carbonate solvents (e.g., DMC, and EMC) suffer from extremely low flash points (<30°C) and boiling points (<130°C), making them prone to ignition under abuse conditions and triggering catastrophic thermal runaway [224, 225, 226]. To address these challenges, extensive efforts have focused on alternative Li salts, functional additives, flame‐retardant solvents, ionic liquids, and organic compound‐based electrolytes to develop intrinsically safer systems.

#### 4.3.1 Strategy I: Alternative Li Salt Design

LiPF_6_ remains the dominant commercial salt because of its balanced ionic conductivity, compatibility with Al collectors, moderate cost, and acceptable toxicity [227]. However, its intrinsic thermal instability represents a critical vulnerability: its decomposition produces HF and PF_5_, which accelerate transition metal dissolution and solvent degradation [228]. Alternative Li salts, such as lithium tetrafluoroborate (LiBF_4_) and lithium bis(trifluoromethanesulfonyl)imide (LiTFSI), offer higher thermal stability [229, 230], but their practical use is hindered by Al corrosion at low concentrations [225].

**FIGURE 5:**
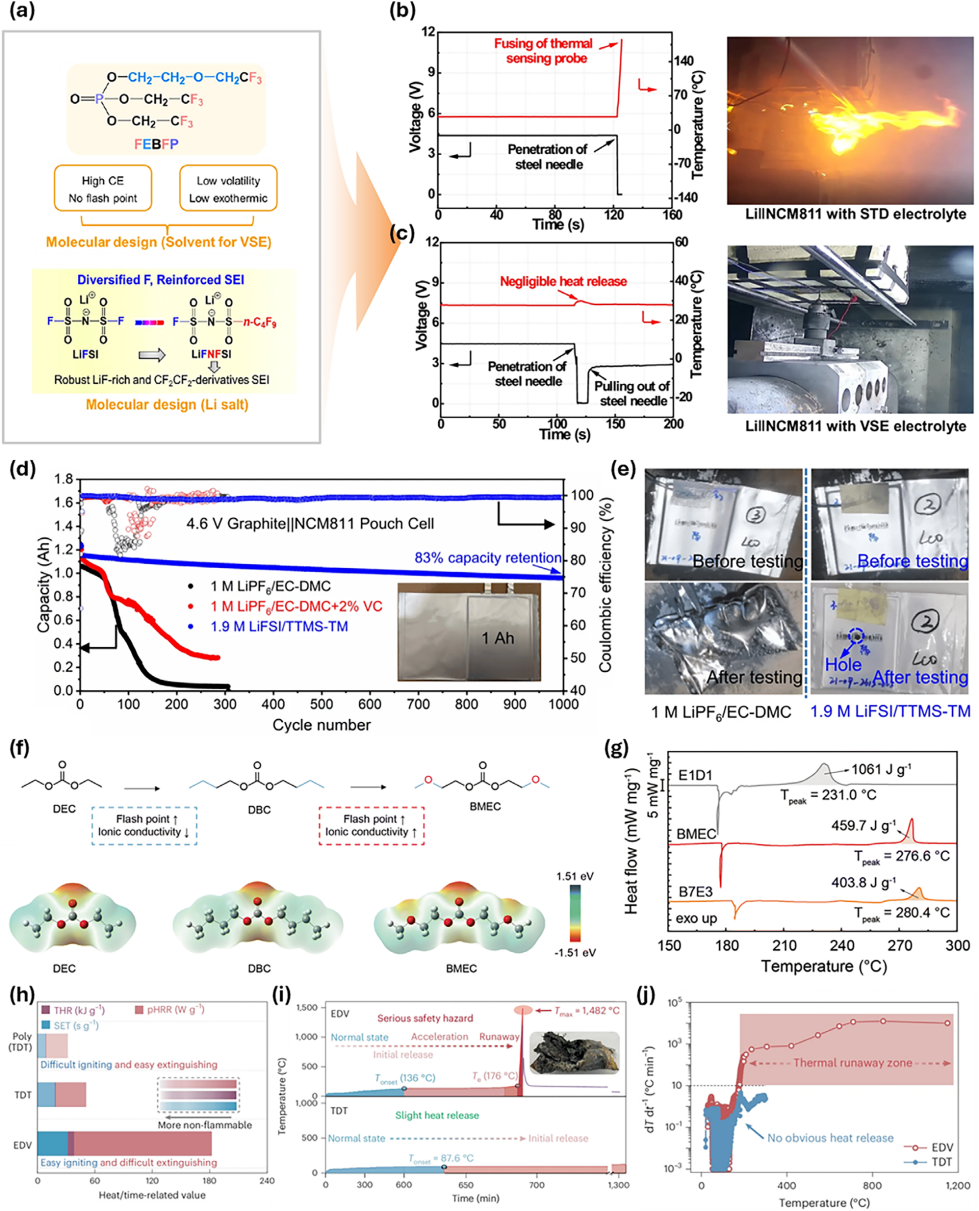
(a) Design strategy of fluorinated phosphate ester solvent and Li salts. Safety tests of fully charged Li||NCM811 pouch cells with (b) conventional and (c) LiFNSI‐based electrolyte (Adapted from Xu et al., Nat. Commun. 15 (2024): 9856) (d) Cycle performance of 1 Ah graphite||NCM811 pouch cells and (e) nail penetration test of 4.55 V charged graphite||NCM811 pouch cells using 1.9 M LiFI in TTM:PC (3:7 v/v%) (Adapted from Zhang et al., Nat. Commun. 14 (2023)). (f) Molecular design strategy and electrostatic potential (ESP) maps of BMEC‐based organic electrolytes. (g) Differential scanning calorimetry of NCM811 cathodes cycled with each electrolyte (Adapted from Lee et al., Energy Environ. Sci. 16 (2023): 2924–2933). (h) Comparison of the self extinguishing time (SET), total head release (THR) and peak heat release rate (pHRR) tested by a cone calorimeter. (i) Temperature‐time curves from ARC tests for LFP||Li pouch cells at a fully charged state. Temperature was increased from 50°C to 300°C with the rate of 5°C min^−1^ and held at each step for 30 min. (j) dT/dt‐T curves of the pouch cell after self‐heat release (Adapted from Yang et al. Nat. Energy 10 (2025) 1493–1502).

Recent studies have focused on salts with improved dissociation and corrosion resistance. For example, lithium (fluorosulfonyl)(nonafluorobutanesulfonyl)imide (LiFNSI) promotes stable SEI formation and prevents Al corrosion. A tailored electrolyte of 0.8 M LiFNSI in fluorinated phosphate ester (FEBFP) prevented thermal runaway in fully charged Li||NCM811 pouch cells during nail penetration testing, while the cell with a standard electrolyte underwent severe explosion and combustion (Figure 5a–c) [231]. Compared with the standard electrolyte (1 M LiPF_6_ in EC:DMC = 1:1 v/v), both the onset temperature of self‐heating reactions associated with SEI decomposition and the self‐sustaining temperature shifted to higher values by approximately 15 °C, indicating enhanced thermal stability. These improvements were attributed to the unique decomposition behavior of LiFNSI salt during initial battery operation, which formed robust SEI and CEI layers enriched with inorganic LiF and fluorinated organic species, such as CF_2_CF_2_‐based components. As these interfacial layers with a high dielectric constant were formed, the overpotential was reduced, and electrolyte decomposition due to side reactions with the electrode was inhibited, especially at high temperatures. Similarly, Chen et al. reported a double‐salt electrolyte (0.8 M LiFSI + 0.2 M lithium difluoro(oxalato)borate (LiDFOB) in FEC:TEGDME, 3:7 v/v) [232]. While LiFSI offers excellent thermal stability (>200°C), it corrodes Al collectors. The addition of LiDFOB formed a robust SEI and passivated the Al films, thereby mitigating corrosion and ensuring high ionic conductivity and thermal stability. This synergistic formulation significantly improved cycling of graphite||NCM622 at 70 °C and significantly retarded the onset of self‐heating behavior.

#### 4.3.2 Strategy II: Controlling of Electrode‐Electrolyte Interface

At elevated voltages and temperatures, the electrolyte decomposition products damage both the SEI and CEI, accelerating structural degradation [233, 234]. Functional additives that regulate the interfacial chemistry thus provide an economical and effective stabilization pathway [232, 233, 235]. Zhang et al. demonstrated that lithium hexamethyldisilazide (LiHMDS) acts as a thermally stable additive that captures acidic species while forming homogeneous CEI layers [236]. Based on the X‐ray photoelectron spectroscopy analysis and Gibbs free energy comparison, it was revealed that LiHMDS is oxidized in Li||NCM811 cell, forming a thin and robust CEI layer. This CEI suppressed the LiPF_6_ decomposition, thereby reducing the formation of acidic byproducts such as HF and inhibiting nickel dissolution from the cathode. Moreover, these reactions promote the formation of the LiF‐rich inorganic SEI layer on the anode, instead of the inhomogeneous layer composed of Li*_x_* PF*_y_*, Li*_x_* PO*_y_* F*_z_*, and NiF_2_. As a result, Li||NCM811 cells with 0.6 wt.% LiHMDS in 1 M LiPF_6_ (EC:EMC:DMC, 1:1:1 v/v) maintained stability over 500 cycles at 60°C, providing good thermal resistance. Chen et al. reported propargyloxytrimethylsilane (PMSL), a siloxane‐based unsaturated additive, which suppressed gas evolution and swelling in graphite||NCM811 pouch cells at 60°C [232]. In‐situ attenuated total reflection‐Fourier transform infrared (ATR‐FTIR) spectroscopy confirmed the formation of insoluble Si–O–R passivation, which mitigated the electrode crosstalk. Li||LiCoO_2_ cells with 1 wt.% PMSL retained ∼90% capacity over 360 cycles at 80°C.

#### 4.3.3 Strategy III: Intramolecular Interaction‐Driven Flame‐Retardant Solvents

Radical quenching, which interrupts combustion by capturing reactive radicals (H• and OH•), has emerged as an effective approach to suppress electrolyte flammability [225, 237, 238]. Fluorine‐ and phosphorus‐containing solvents are known to be particularly promising [229, 239]. Fluorinated electrolytes not only scavenge combustion radicals (via F•) [240]. but also benefit from the electron‐withdrawing effect of F atoms, enhancing the oxidative stability under high voltage [241]. Recent examples include ethoxy(pentafluoro)cyclotriphosphazene (PFPN), methyl difluoroacetate (MDFA), and 2,2,2‐trifluoroethyl trifluoromethanesulfonate (TTMS) [242, 243, 244]. An et al. developed a nonflammable electrolyte consisting of 1 M LiPF_6_ dissolved in 2,2,2‐trifluoroethyl acetate (TFA) and propylene carbonate (PC) (3:7 v/v) [240]. The electron‐withdrawing fluorine atoms in the alkoxy chain of TFA strengthened the C─O bonds, imparting high oxidative stability above 5 V (vs. Li/Li^+^). In addition, the high electronegativity of fluorine allows for efficient scavenging of reactive radicals generated during combustion, thereby suppressing fire propagation. Benefiting from these properties, the TFA‐based electrolyte enabled ∼82% capacity retention over 400 cycles at 45°C in 730 mAh g^−1^ graphite||NCM811 pouch cells. Zhang et al. reported a flame‐retardant electrolyte composed of TTMS and 2,2,2‐trifluoroethyl methanesulfonate (TM) (1:2 v/v) with a 1.9 M LiFSI [245]. This formulation delivered ∼83% capacity retention after 1000 cycles in 1‐Ah graphite||NCM811 pouch cells and effectively mitigated thermal runaway up to at least 170°C, as validated by ARC tests (Figure 5d,e).

Phosphorus‐based electrolytes generate P‐centered radicals (e.g., PO•, HPO•, and HPO_2_•) that effectively quench the combustion [242]. Phosphate solvents such as trimethyl phosphate (TMP) and triethyl phosphate (TEP) have been extensively studied as flame‐retardant candidates because of their intrinsic nonflammability, low viscosity, and high oxidative stability [246, 247, 248]. However, despite these advantages, phosphorus‐containing electrolytes suffer from the inability to form a stable SEI on the anode surface, which results in graphite exfoliation and continuous electrolyte decomposition during the initial charging [247]. To overcome these limitations, Zheng et al. developed a molecular design strategy by introducing fluorinated moieties [249]. They formulated an electrolyte composed of 0.95 M LiFSI dissolved in a mixture of tris(2,2,2‐trifluoroethyl) phosphate (TFEP) and 2,2,2‐trifluoroethyl methyl carbonate (FEMC) (1:3 v/v). This formulation facilitated the formation of a stable SEI layer via ring‐opening polymerization, facilitated by the presence of phosphorus. Importantly, the phosphorus center plays a dual role: (1) the P atom in TFEP undergoes nucleophilic attack by oxygen species on the oxygen‐rich surface of the transition metal cathodes, triggering polymerization and forming a robust CEI; and (2) the phosphorus moiety serves as a hydrogen radical scavenger, thereby suppressing combustion chain reactions. Owing to these synergistic effects, the dual‐functional electrolyte effectively suppressed electrolyte oxidation and transition‐metal dissolution, achieving ∼80.1% capacity retention after 500 cycles in Li||NCM111 cells. In a complementary approach, Meng et al. demonstrated that incorporating butenoxycyclotriphosphazene (BCPN) monomers into the electrolyte enhances the non‐flammability and contributes to the Li^+^ solvation sheath [250]. This not only improves the oxidative stability of the electrolyte, but also leads to superior electrochemical performance in Li||NCM811 pouch cells.

#### 4.3.4 Strategy IV: Intermolecular Interaction‐Driven Electrolyte Engineering

In addition to chemical flame suppression, recent strategies have leveraged strong intermolecular interactions to suppress the volatility and enhance the thermal resilience [251]. Ionic liquids and high‐temperature organic electrolytes have been explored for their ability to reduce flammability, stabilize interphases, and extend the operating temperature window [252, 253].

Ionic liquids offer wide electrochemical windows, tunable viscosity, and high thermal stability with strong cation–anion interactions, yielding inherently low volatility and flammability [254, 255]. For example, a 0.8 M Pyr_14_FSI–0.2 M LiTFSI electrolyte balanced ionic conductivity and viscosity, enabling >200 stable cycles at 60°C in Li||NCM811 cells by promoting a dense SEI and mitigating electrolyte‐driven degradation [254]. Similarly, Sun et al. demonstrated a nonflammable [EMIm]‐based ionic liquid with excellent moisture tolerance and thermal stability [255]. The [EMIm]^+^ cation, with a delocalized positive charge, reduced viscosity and enhanced conductivity, whereas a high LiFSI concentration altered the solvation to favor Li^+^ reduction and suppress cation decomposition at low potentials. The addition of sodium bis(trifluoromethanesulfonyl)imide (NaTFSI) further promoted the formation of LiF–NaF hybrid interfaces and provided electrostatic shielding, suppressing dendrite growth and stabilizing both CEI and SEI layers. Notably, thermogravimetric analysis (TGA) and flammability tests confirmed that the electrolyte remained thermally stable without noticeable decomposition from 30°C to 300°C, representing a significant improvement over volatile EC/DMC‐based electrolytes. These results suggest that ionic‐liquid‐based electrolyte design can improve high‐temperature safety by reducing electrolyte volatilization, suppressing interfacial instability, and mitigating thermal‐runaway‐prone degradation while addressing the ionic‐transport limitations typically associated with high viscosity.

High‐temperature organic electrolytes also benefit from enhanced solvent–solvent interactions that increase the flash and boiling points, thereby suppressing ignition. Lee et al. reported that dibutyl carbonate (DBC) increased the flash point via stronger intermolecular interactions, whereas the addition of bis(2‐methoxyethyl) carbonate (BMEC) improved the ionic conductivity and vaporization enthalpy [253]. A BMEC:EC (7:3 v/v) electrolyte with 1 M LiPF_6_ achieved ∼91.4% capacity retention after 500 cycles in 1 Ah graphite||NCM811 cells (Figure 5f,g). Compared with the conventional electrolyte (1 M LiPF_6_ in EC/DEC (1:1 v/v) with 3 vol % FEC; E1D1), the BMEC‐containing electrolyte delayed the onset temperature of exothermic reactions in the fully charged state by at least 45 °C and reduced the total heat release by more than 50%. BMEC‐based electrolytes also generated less flammable gas under thermal abuse conditions. These findings indicate that optimizing solvent–solvent interactions in BMEC‐based electrolytes can mitigate rapid temperature rise and suppress thermal runaway by improving thermal stability, reducing heat release, and lowering flammable‐gas generation.

#### 4.3.5 Strategy V: Thermoresponsive Electrolytes

Thermoresponsive electrolytes have recently emerged as active safety electrolytes that can alter their physicochemical properties in response to abnormal temperature elevation. Unlike conventional flame‐retardant or non‐flammable electrolytes, which mainly reduce fuel availability or combustion intensity, thermoresponsive electrolytes are designed to trigger a temperature‐dependent transition before separator collapse or severe ISCs occur. This transition may involve in situ polymerization, liquid‐to‐solid conversion, gelation, viscosity increase, or temperature‐induced solvation reconfiguration. As a result, thermoresponsive electrolytes can suppress thermal‐runaway‐driving processes by reducing solvent mobility, limiting electrode–electrolyte contact, blocking ion transport through damaged regions, and delaying short‐circuit‐induced heat accumulation [256, 257, 258].

Early thermoresponsive electrolyte designs demonstrated that thermally activated polymerization can improve safety without fully compromising electrochemical operation at normal temperatures. Jiang et al. reported a thermoresponsive electrolyte for Li‐metal batteries in which temperature‐triggered polymerization immobilized liquid electrolyte components and improved high‐temperature compatibility with electrodes [256]. This concept established an important design principle: the electrolyte should maintain sufficient ionic conductivity during normal cycling, but rapidly transition into a less mobile and more thermally stable state under overheating conditions. However, the activation temperature, polymerization heat, volume change, and compatibility with commercial separators must be carefully controlled because delayed response or excessive heat release during polymerization can undermine the intended safety function.

Recent studies have further refined this concept by designing thermoresponsive electrolytes that balance cycling stability and thermal safety. Gu et al. introduced a thermoresponsive ether‐based electrolyte using 1,3,5‐trioxane in a LiFSI/tetrahydrofuran (THF) system [257]. At elevated temperature, 1,3,5‐trioxane triggered cationic ring‐opening polymerization of THF, generating thermally stable ether‐based polymeric species while also reconfiguring the Li^+^ solvation structure. This dual effect improves interfacial stability and enables wide‐temperature operation of Li‐metal batteries, indicating that thermoresponsive electrolyte design can simultaneously regulate ion transport, interfacial chemistry, and thermal response.

A more direct thermal‐shutdown strategy was demonstrated by Yang et al. using an ultrafast thermoresponsive electrolyte composed of 2 M LiTFSI, 0.5 M LiNO_3_, and 10 mM LiPF_6_ in a mixed solvent of triethylene glycol divinyl ether (TEGDVE), 1,2‐difluorobenzene (1,2‐DFB), and triglyme at a 3:2:5 v/v/v ratio [258]. In this system, LiPF_6_ initiates cationic polymerization of TEGDVE at elevated temperature, rapidly converting the electrolyte from a liquid to a solid‐like state before separator melting. The resulting poly(TEGDVE)‐based phase acts as a physical and thermal barrier that limits ISCs and suppresses thermal runaway. The minor volume change of approximately 0.8% during solidification also reduces the risk of mechanically induced short circuits during the liquid‐to‐solid transition. ARC and cone calorimeter tests showed that this thermoresponsive transition effectively suppressed heat release and prevented thermal runaway in fully charged pouch cells (Figure 5h–j).

These recent studies suggest that thermoresponsive electrolytes can help the conventional trade‐off between electrochemical performance and thermal safety by remaining conductive during normal operation while activating a protective response under overheating conditions. Nevertheless, several challenges remain. The activation temperature must be matched to the separator shutdown or melting window, the response must occur faster than short‐circuit propagation, and the polymerized or gelled product should maintain dimensional stability without generating excessive heat or gas. In addition, scalability, long‐term storage stability, compatibility with high‐voltage cathodes, and reproducibility in large‐format cells should be further validated. Therefore, thermoresponsive electrolytes represent a promising active electrolyte strategy, but their practical implementation requires careful coordination among response temperature, reaction kinetics, electrochemical stability, and cell‐level safety design.

#### 4.3.6 Strategy VI: Solid‐State Electrolytes

Replacing flammable organic liquid electrolytes with solid‐state electrolytes (SSEs) represents a more fundamental strategy for improving battery safety. Conventional liquid electrolytes can promote thermal runaway through leakage, volatilization, combustion, and separator‐related failure under abusive conditions. In contrast, SSEs can reduce several electrolyte‐driven failure triggers by serving as ion‐transport media and, in many cell architectures, mechanically robust separators. Their low volatility, reduced flammability, high mechanical rigidity, and relatively wide electrochemical stability windows can mitigate electrolyte‐fueled combustion and help suppress dendrite‐induced ISCs [259, 260].

SSEs are generally classified into sulfide‐, oxide‐, halide‐, and polymer‐based systems. Sulfide‐based SSEs are attractive because of their high room‐temperature ionic conductivity and mechanical deformability, which facilitate intimate electrode–electrolyte contact and scalable processing. Oxide‐based SSEs, including garnet‐ and sodium superionic conductor (NASICON)‐type materials, generally provide high chemical and thermal stability, although their rigid solid–solid interfaces often require careful interfacial engineering. Halide‐based SSEs have recently attracted attention owing to their oxidative stability and favorable compatibility with high‐voltage cathodes. Polymer‐based SSEs offer flexibility, facile processing, and improved electrode–electrolyte interfacial contact, although their ionic conductivity and thermal stability still require further optimization. These complementary characteristics make SSEs important components of next‐generation battery architectures aimed at improving both safety and energy density [259, 260, 261].

Nevertheless, replacing liquid electrolytes with SSEs does not by itself eliminate thermal runaway risk. Recent studies have shown that solid‐state lithium batteries can still undergo severe thermal failure through interfacial instabilities, exothermic reactions between electrodes and SSEs, dendrite penetration, hazardous gas generation, and localized heat accumulation within full‐cell architectures. These risks are particularly relevant to sulfide‐based all‐solid‐state batteries, where highly delithiated layered oxide cathodes and Li metal anodes can react with sulfide SSEs under thermal abuse. Therefore, although SSEs provide an important route toward intrinsically safer batteries, thermal safety in solid‐state systems must still be engineered through coordinated advances in electrolyte chemistry, interface stabilization, electrode architecture design, and cell‐level thermal management [22, 262].

The electrolyte strategies discussed in this section mitigate thermal runaway through different chemical and physicochemical pathways. Alternative salts and fluorinated electrolyte systems primarily stabilize electrode–electrolyte interphases and reduce exothermic reactions at reactive electrode surfaces. Flame‐retardant solvents and high‐temperature organic electrolytes suppress ignition and reduce heat or gas release by increasing thermal stability, vaporization enthalpy, or nonflammability. Ionic liquids provide low volatility and high thermal stability, although their practical use can be limited by viscosity, cost, and compatibility with high‐loading electrodes. Thermoresponsive electrolytes provide an active safety response by changing their state or solvation structure under abnormal temperature elevation while maintaining normal cycling operation. Because these strategies differ in interfacial stability, thermal response, cycling performance, cost and scalability, Table 4 compares their representative safety indicators, cycling performance, practical readiness, and key limitations.

**TABLE 4:** Comparative summary of electrolyte strategies for balancing cycling stability and thermal safety.

Strategy	Representative formulation or approach	Main safety mechanism	Representative thermal safety indicator	Representative cycling performance	Cost/scalability	Key limitation
Alternative salt and fluorinated salt design	LiFNSI or fluorinated salt in fluorinated phosphate ester solvent	Forms robust SEI/CEI layers, suppresses exothermic interfacial reactions, reduces flammability	Nail penetration safety improvement, delayed self‐exothermic onset by ∼15 °C, reduced combustion severity	Stable operation in high‐energy pouch‐cell configurations depending on formulation	Moderate, but salt cost and synthesis route matter	Cost of fluorinated salts, compatibility with high‐loading electrodes, possible viscosity and wetting issues
Interface‐forming additives	FEC, VC, LiNO_3_, LiDFOB, fluorinated or boron‐containing additives	Forms protective SEI/CEI, suppresses gas evolution, reduces electrolyte decomposition at reactive surfaces	Increased onset temperature of interfacial reactions, reduced gas generation, improved DSC/ARC response	Often improves cycle retention when additive concentration is optimized	High, because low additive content is compatible with existing manufacturing	Narrow optimal concentration, additive depletion, side reactions at high voltage
Flame‐retardant solvents or additives	Phosphate, phosphazene, fluorinated carbonate, or fluorinated phosphate ester systems	Reduces flammability, interrupts radical combustion, lowers heat release	Reduced SET, THR, pHRR, ignition probability, and flame propagation	Can maintain cycling when solvation and interphase chemistry are optimized	Moderate, depending on solvent cost and viscosity	Possible conductivity loss, viscosity increase, poor low‐temperature performance, compatibility with graphite
Ionic liquids	Pyrrolidinium‐, imidazolium‐, or FSI/TFSI‐based ionic liquid electrolytes	Low volatility, high thermal stability, nonflammability, strong ion association	TGA stability up to high temperature, suppressed flammability, reduced electrolyte evaporation	Can enable elevated‐temperature cycling when viscosity and interphase chemistry are balanced	Limited to moderate, due to high cost and viscosity	High viscosity, lower rate capability, cost, wetting difficulty, large‐format validation
High‐temperature organic electrolytes	High‐flash‐point carbonate or ether‐carbonate solvent systems such as BMEC‐based electrolytes	Increases flash point, boiling point, vaporization enthalpy, and thermal stability	Exothermic onset delayed by ≥45 °C, total heat release reduced by >50%, lower flammable‐gas generation	∼91.4% capacity retention after 500 cycles in 1 Ah graphite	—	NCM811 cells, depending on formulation

### 4.4 Separator

The separator serves as a critical passive safety barrier by physically isolating the anode and cathode. However, conventional materials are thermally unstable and mechanically fragile at elevated temperatures, risking ISCs [130, 263]. To enhance the safety feature, strategies should include ceramic coatings, multilayer structures, electrospun fiber membranes, high‐thermal‐resistance polymer composites, and stimuli‐responsive smart materials [264, 265, 266]. These approaches improve thermal and mechanical robustness while maintaining compatibility with advanced electrodes and electrolytes.

#### 4.4.1 Ceramic‐Coated Separator (CCS)

**FIGURE 6:**
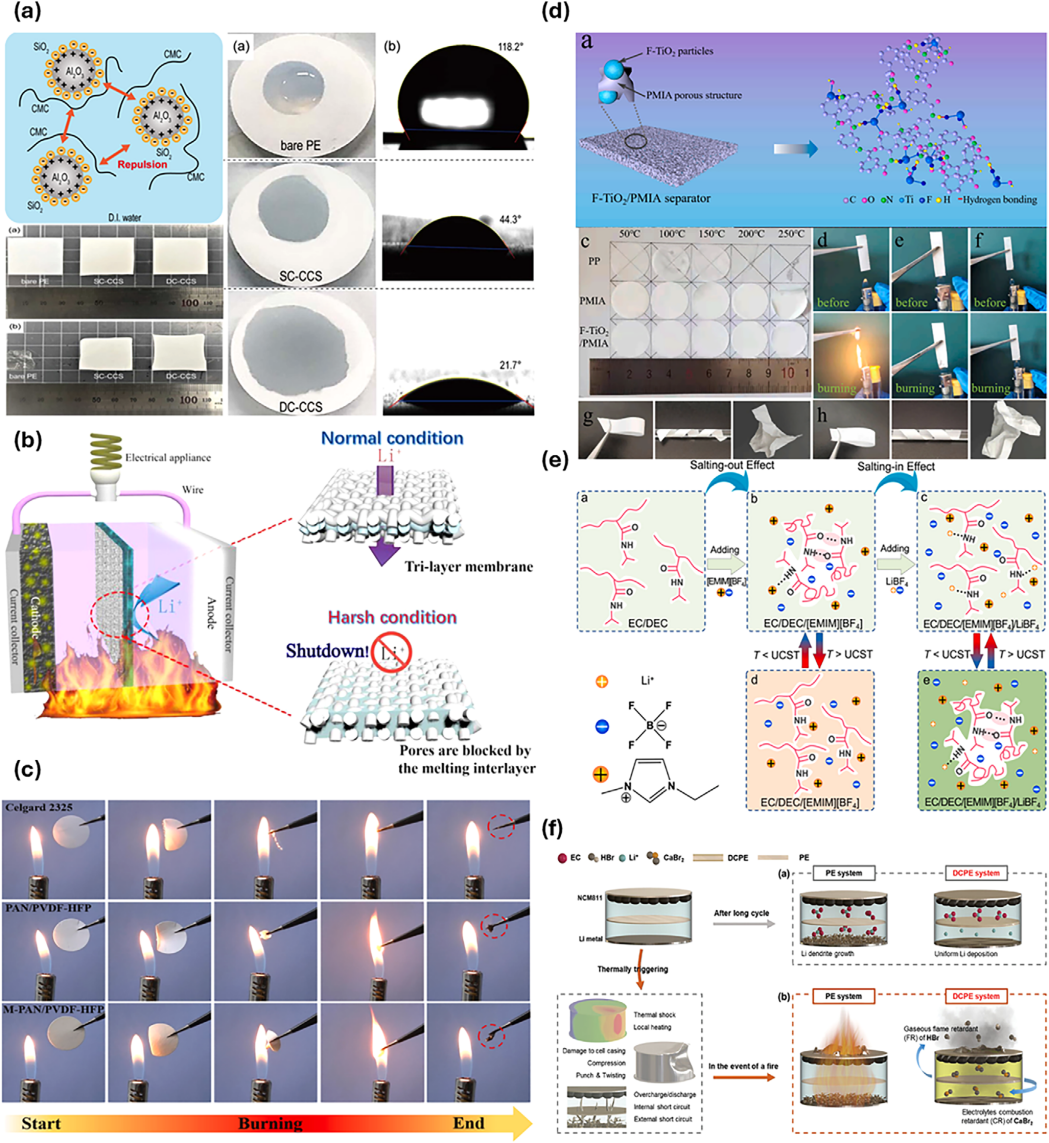
(a) Dual‐coating ceramic‐coated separator (DC‐CCS): structure, thermal shrinkage, and wettability comparison. (Adapted from Kennedy et al., 2022) (b) Tri‐layer nonwoven separator with thermal shutdown function (Adapted from Li et al., 2018) (c) Combustion tests of Celgard 2325, PAN/PVDF‐HFP and M‐PAN/PVDF‐HFP separators. (Adapted from Guo et al., 2023) (d) PMIA‐based composite separator anchored with fluorine‐functionalized TiO_2_ particles via directed assembly: fabrication, thermal stability, and electrochemical performance (Adapted from Hu et al., 2023) (e) Thermal‐triggered gating behavior of PNIPAM‐modified separator in ionic liquid electrolyte. (Adapted from Jiang et al., 2024) (f) Mechanistic schematic of DBDPE and CaO‐coated PE separator (DCPE) under normal and abuse conditions compared with PE separator. (Adapted from Yang et al., 2024).

Ceramic coatings, which are widely adopted for practical scalability, reinforce the thermal and mechanical integrity by suppressing shrinkage and enhancing puncture resistance [263]. Conventional polyolefin separators suffer severe dimensional loss above 100°C, increasing the risk of ISCs, while insufficient tensile or puncture strength exacerbates failure under mechanical stress or dendrite penetration [267]. Incorporating ceramic fillers such as Al_2_O_3_, SiO_2_, and ZrO_2_ forms a rigid and thermally stable scaffold that sustains the separator structure under abusive conditions [268, 269, 270, 271]. The degree of reinforcement depends on both the material and coating configuration [261]. For example, Al_2_O_3_ coatings with high hardness and chemical stability are widely used in commercial CCSs. Kennedy et al. showed that a uniformly dispersed Al_2_O_3_ layer limits shrinkage to < 2% at 150°C while enhancing puncture resistance (Figure 6a) [268]. Gong et al. reported additional benefits including increased tensile strength and improved electrolyte wettability [269]. SiO_2_ coatings, particularly nanoporous sponge‐like layers, combine flexibility with rigidity; Ding et al. demonstrated that a SiO_2_‐based PP separator retains morphology under high C‐rate fast charging due to its interconnected porosity and thermal insulation [270]. ZrO_2_, with superior thermal resistance and dendrite‐blocking capability, supports high‐temperature applications: ZrO_2_@polyimide‐coated separators show negligible shrinkage up to 160 °C and enhanced puncture strength, enabling stable cycling under mechanical abuse [271]. Hybrid designs combining Al_2_O_3_, SiO_2_ or ZrO_2_ with polymers further synergize these benefits, extending separator durability under aggressive thermal and mechanical regimes. Gogia et al. reported that ultra‑thin, binder‑free coatings incorporating Al_2_O_3_ nanoparticles form a continuous ceramic framework that suppresses thermal shrinkage and reinforces puncture resistance under elevated temperatures [272].

#### 4.4.2 Structurally Engineered Separators

Beyond surface coatings, structural engineering of separators can provide intrinsic thermal, mechanical, and electrochemical robustness [112]. Multilayer architectures, such as PP/PE/PP tri‐layers, leverage melting‐point gradients for passive thermal shutdown: the lower‐melting PE core (∼125–135 °C) collapses under excessive heat to block ion transport, while higher‐melting PP outer layers (∼165°C) preserve structural integrity and prevent ISCs, ensuring physical separation of the electrodes [129]. Similarly, co‐extruded multilayer PP/PE separators have shown well‐defined shutdown at 132 °C with minimal impact on electrochemical performance [273]. Fiber‐based trilayer designs (PP/PE/PP) further enhance the mechanical strength and thermal response, making them suitable for high‐energy‐density cells (Figure 6b) [274].

Next‐generation separators integrate asymmetric structures, gradient porosity, and core–shell fiber morphologies to combine thermal shutdown, dendrite suppression, and ion transport control [275, 276, 277]. Xing et al. fabricated nanofibrous separators with vertical orientation gradients, tightly packed fibers provided mechanical rigidity, whereas loosely arranged fibers on the opposite side improved the electrolyte wettability and ion mobility [275]. The resulting layer‐by‐layer electrospun architecture preserved the structural integrity at elevated temperatures and improved the ionic conductivity and cycling stability under high‐rate operation. Gao et al. reported a core‐shell separator fabricated via coaxial electrospinning with a polyacrylonitrile (PAN) core and a poly(vinylidene fluoride) (PVDF) shell. In multilayer films, the PAN core imparts a high tensile strength and thermal stability, whereas the PVDF shell enhances the electrolyte affinity and ionic conductivity, collectively reducing the interfacial impedance and improving the electrochemical performance under thermal stress [276]. In another approach, the same authors developed a Janus‐type hybrid membrane with PAN on one side and poly(vinylidene fluoride‐co‐hexafluoropropylene) (PVDF‐HFP) on the other side joined by a mussel‐inspired adhesive interlayer (Figure 6c) [277]. This asymmetric architecture enables directional electrolyte transport, suppresses dendrite growth at the anode, and maintains cathode wetting, resulting in enhanced cycle life and safety under high‐voltage and high‐temperature conditions. These structurally engineered separators exemplify the transition from passive barriers to multifunctional protective layers, where tailored internal architectures, such as fiber orientation, compositional asymmetry, or nanoscale encapsulation, simultaneously enhance the thermal stability, mechanical robustness, and electrochemical performance.

#### 4.4.3 Electrospun and Fiber‐Based Separators

Electrospun and fiber‐based membranes combine high porosity, mechanical compliance, and thermal resilience, making them promising candidates for next‐generation batteries [265, 278]. Fabricated via electrospinning, these nonwoven mats feature tunable fiber morphology, diameter, and nanoscale pore connectivity, yielding 3D architectures that support ion transport while maintaining structural flexibility and strength [279, 280]. Their material‐level tunability allows for the use of high‐performance polymers with intrinsic thermal resistance and mechanical rigidity [281, 282, 283, 284, 285, 286]. For example, poly(phenylene sulfide) electrospun separators with rigid–flexible architectures and a semi‐crystalline form withstand temperatures up to 300°C without shrinkage, maintaining mechanical integrity and electrolyte compatibility [281]. Electrospun polyimide (PI) membranes similarly retain morphology and porosity above 300°C, outperforming alternatives such as PVDF, polysulfone (PSU), and PA6(3)T in thermal endurance [282]. The superior behavior stems from inherently high thermal stability of PI due to its aromatic imide backbone, combined with the mechanically interlocked fiber network created by electrospinning which resists collapse under thermal stress. PVDF and its copolymers (e.g., PVDF‐HFP) offer moderate thermal resistance (180–200°C) with high polarity and electrochemical stability, providing balanced performance for practical operation [283].

Beyond polymer selection, embedding functional fillers, ceramic particles or multi‐element flame retardants, directly into the fiber matrix can further enhance safety and electrochemical performance [284, 285]. A representative example is the incorporation of Al_2_O_3_ nanoparticles into PI frameworks, which reduces thermal shrinkage and maintains electrochemical stability by acting as a thermally inert scaffold [284]. Deng et al. incorporated phosphorus‐nitrogen‐sulfur (P‐N‐S) flame retardants into a gel‐type poly‐m‐phenyleneisophthalamide (PMIA) electrospun membrane, imparting self‐extinguishing properties and an increased oxygen index, thereby enabling the active suppression of combustion during thermal runaway [285]. Meanwhile, Hu et al. anchored fluorinated TiO_2_ nanoparticles onto PMIA fibers, which enhanced electrolyte wettability, ionic conductivity, and voltage stability, while preserving dimensional integrity up to 250°C (Figure 6d) [286].

Collectively, these electrospun composite separators demonstrate how synergistic polymer–filler interactions can be finely tuned to provide both passive thermal protection and active mitigation of hazardous failure modes, supporting high‐energy and high‐voltage LIB applications [287].

#### 4.4.4 Functional Separators

Beyond structural reinforcement and composite integration, the next frontier in separator design is smart stimuli‐responsive systems that actively respond to changes in the cellular environment [288, 289]. These functional separators not only withstand thermal and mechanical stress, but also autonomously mitigate hazards by shutting down ion transport, self‐healing structural defects, or suppressing thermal propagation [290, 291]. In particular, thermally responsive separators are key innovations that dynamically alter the ionic permeability or internal structure in response to temperature [292, 293]. For example, polymers with thermogating behavior, such as poly(N‐isopropylacrylamide) (PNIPAM), can reversibly block ionic channels at elevated temperatures. Jiang et al. grafted PNIPAM onto a polypropylene substrate, where chain collapses at the upper critical solution temperature halted ion transport, and subsequent reswelling restored conductivity (Figure 6e) [292]. This reversible gated ion transport, validated by electrolyte diffusion and ionic conductivity measurements, demonstrates the potential for autonomous self‐protection in lithium‐based systems. In complementary approaches employing phase‐change materials (PCMs) for thermal regulation, Huang et al. encapsulated paraffin wax within electrospun PAN fibers to absorb heat via the latent heat of fusion, delaying the temperature rise, while an metal organic framework (MOF)/black phosphorus coating stabilized the nanofiber mechanically and chemically by suppressing lithium polysulfide migration and dendrite growth, achieving over 1000 cycles in Li–S cells [293].

Smart separators also function as chemical stabilizers by releasing flame retardants or scavenging hazardous gases. Yang et al. designed a separator coated with a decabromodiphenyl ethane (DBDPE)–calcium oxide (CaO) nanocomposite (Figure 6f) [294]. Under thermal abuse, this coating activates a dual‐phase flame‐retardant mechanism: DBDPE decomposes to release hydrogen bromide (HBr), which quenches flames in the gas phase, while CaO reacts with HBr to form CaBr_2_, providing condensed‐phase flame suppression. This dual action mitigates flame propagation and suppresses internal electrolyte decomposition. In addition, the nanocomposite layer improves electrochemical performance by enhancing ionic conductivity, increasing the Li‐ion transference number, and reducing polarization and dendrite formation, thereby promoting more stable cycling and extending battery safety under high‐temperature and high‐voltage conditions. Alternatively, metal–organic framework (MOF)‐based separators can provide a preventive strategy against battery failure by rapidly and selectively adsorbing hazardous species such as HF and O_2_, leveraging their high surface area and tunable pore chemistry [295, 296, 297]. For example, Li et al. demonstrated a UiO‐66‐F@PP coated separator (MOF coating on polypropylene) that effectively captures gas and moisture species while enhancing thermal and electrochemical stability in high‐voltage cells [298]. Similarly, Kim et al. applied sulfonated UiO‐66/Nafion hybrids to form a proton‐conductive, high‐affinity layer that not only traps moisture‐borne HF and oxygenated species, but also mitigates polysulfide shuttling in Li–S cells, resulting in a prolonged cycle life and improved thermal resilience [299].

The separator strategies discussed in this section mitigate thermal runaway by maintaining physical separation between electrodes while preserving ion transport under normal operation. Ceramic‐coated separators mainly enhance dimensional stability and thermal resistance, while multilayer shutdown separators are designed to block ion transport before severe ISCs occur. Electrospun, composite, and high‐temperature polymer separators can further improve thermal tolerance, puncture resistance, and electrolyte wettability. Smart or stimuli‐responsive separators add functions such as thermal shutdown, flame retardancy, or ion‐transport regulation, although their scalability and long‐term reliability require further validation. Because separator safety depends not only on thermal stability but also on mechanical deformation, shrinkage behavior, puncture resistance, shutdown response, ionic conductivity, and electrolyte uptake, it cannot be evaluated using a single metric. Table 5 therefore compares representative separator strategies using criteria that reflect both thermal protection and electrochemical compatibility, including dimensional stability, mechanical integrity, shutdown behavior, ionic conductivity, scalability, and key limitations.

**TABLE 5:** Comparative summary of separator strategies for suppressing shrinkage, internal short circuits, and thermal propagation.

Strategy	Representative material or design	Main safety function	Representative thermal or mechanical indicator	Electrochemical compatibility	Cost/scalability	Key limitation
Ceramic‐coated polyolefin separator	Al_2_O_3_, SiO_2_, boehmite, ZrO_2_, or other ceramic coating on PE/PP separator	Suppresses thermal shrinkage, improves wettability, maintains electrode separation	Reduced shrinkage at elevated temperature, improved dimensional stability, higher puncture resistance	Generally good, often improves electrolyte uptake	High to moderate, already used in some commercial formats	Coating adhesion, particle shedding, thickness increase, process cost
Multilayer shutdown separator	PE/PP/PE or PP/PE/PP multilayer separator	Pore closure blocks ion transport before catastrophic ISC	Shutdown near PE melting region, structural support from PP layer at higher temperature	Good if shutdown does not occur during normal high‐power operation	High, compatible with existing separator production	Limited protection after full melting, possible shrinkage under severe abuse
High‐temperature polymer separator	PI, aramid, PBI, PVDF‐HFP composite, or other heat‐resistant polymer	Maintains mechanical integrity above polyolefin softening temperature	Low shrinkage at high temperature, improved thermal dimensional stability	Depends on porosity and electrolyte wettability	Moderate to limited	Higher material cost, processing complexity, possible lower shutdown function
Electrospun or porous fiber membrane	Electrospun polymer or ceramic/polymer fiber network	Improves porosity, electrolyte uptake, thermal tolerance, and mechanical buffering	High electrolyte uptake, enhanced dimensional stability, improved puncture resistance	Often favorable ionic conductivity due to high porosity	Limited to moderate	Thickness control, mechanical robustness, scale‐up of electrospinning
Flame‐retardant or functional separator	Separator containing phosphorus, ceramic, radical scavenger, or flame‐retardant functional groups	Reduces flame propagation and suppresses combustion near separator failure	Lower flammability, delayed ignition, reduced flame spread	Must maintain ion transport and electrolyte compatibility	Moderate to limited	Additive leaching, impedance increase, durability under cycling
Smart or stimuli‐responsive separator	Thermally responsive, redox‐responsive, or self‐shutdown separator	Provides active ion‐transport blocking or functional response under abnormal conditions	Temperature‐triggered shutdown, resistance increase, or functional transition	Promising but system‐dependent	Currently limited	Response reproducibility, long‐term stability, large‐format validation

## 5 Cell and Structural Approaches for Safety Enhancement

Large‐scale battery systems, such as EVs and ESS, are hierarchically assembled, where individual cells are grouped into modules for mechanical protection, and the modules are integrated into packs with a BMS and cooling unit [300]. Achieving high safety requires a comprehensive understanding of both the cell‐ and system‐level designs. This section highlights the representative cell formats and associated safety engineering strategies, followed by system‐level approaches.

### 5.1 Cell‐Level Design

Cell formats are generally classified by geometry and casing material: (1) *Cylindrical cells* feature a jellyroll structure enclosed in steel, offering high mechanical stability and compatibility with safety devices such as positive temperature coefficient (PTC) thermistors, current interrupt devices (CIDs), and safety valves [301, 302]. However, their drawbacks include reduced packing efficiency due to inter‐cell voids and lower gravimetric energy density from the steel case; (2) *Pouch cells* are composed of stacked electrodes sealed in an aluminum–plastic laminate, enabling design flexibility, high packing efficiency, and rapid multi‐directional electrolyte wetting [303, 304]. However, they are susceptible to pressure‐induced deformation and require complex processing steps such as degassing, resealing, and precision welding to ensure both mechanical and electrical stability; and (3) *Prismatic cells* adopt either stacked or wound electrodes within a rigid rectangular casing, combining the mechanical robustness of cylindrical cells with the packing efficiency of pouch cells [305, 306]. However, the electrode–housing mismatch creates internal voids and dead volume, whereas the heavy casing limits the energy density [307].

#### 5.1.1 Cylindrical Cells

**FIGURE 7:**
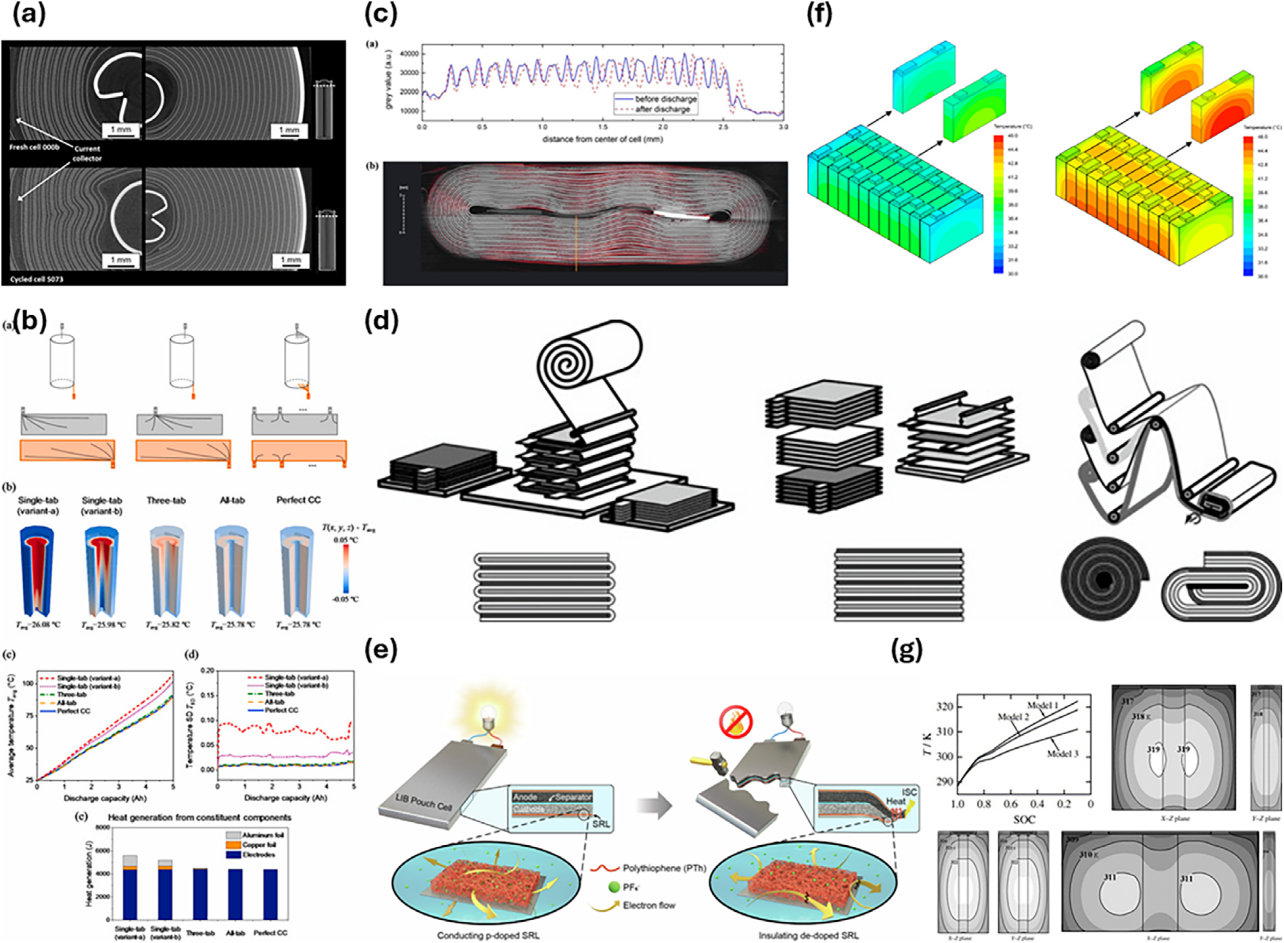
(a) High‐resolution CT cross‐sections of a fresh (top) and cycled (bottom) cell, taken near the cell top (locations indicated in inserts). Left panels show the region between the Al current collector and the cell center, while right panels show the opposite side. (Adapted from Pfrang, A. et al., 2019) (b) Model predictions illustrating the effect of cell tab design on cell performance. (Adapted from Shen, L. et al., 2021) (c) Close‐up longitudinal sections of the graphite overhang region (from one edge of the cell) taken from the CT scans before and after overdischarge, respectively. (Adapted from Bond, T. et al., 2017) (d) Representation of stacking (left), z‐folding (middle) and winding processes (right) for production of electrode–separator composites and their resulting shape. (Adapted from Antje Schilling et al., 2016) (e) A schematic diagram of a thermal runaway prevention mechanism in practical batteries employing a polythiophene (PTh)‐based safety reinforced layer (SRL). (Adapted from Song, I. T. et al., 2024) (f) Temperature distribution on the module surface, 1C discharge (left); 2C discharge (right). (Adapted from Mohsen, A. et al., 2020) (g) Numerically obtained transient variations (left, top) and temperature distribution of highest temperature in Model 1‐ (left, bottom), 2‐ (right, top), and 3‐type (right, bottom) prismatic batteries. (Adapted from Inui, Y. et al., 2007).

Thermal runaway in cylindrical cells typically originates from jellyroll collapse triggered by structural, electrochemical, and mechanical stresses (Figure 7a) [308, 309]. To mitigate ISCs caused by edge lithium plating, anode overhangs are commonly employed to maintain consistent electrode alignment. However, this design feature also introduces bending stress in the innermost anode during winding process. Therefore, jellyroll collapse is often linked to buckling‐driven structural failure, which is initiated in the inner layers where the mechanical, electrochemical, and structural stresses converge. Specifically, Li^+^ intercalation and the associated irreversible expansion generate hoop and radial compressive stresses that accumulate in the core. Analytical models based on the Euler buckling and hoop stress confirmed that the innermost region had the lowest critical stress threshold, rendering it structurally vulnerable. This susceptibility is further exacerbated in the absence of mechanical reinforcements, such as center pins, which provide countersupport against radial collapse [308, 309, 310]. In addition, a non‐uniform current distribution can cause spatially heterogeneous thermal gradients, with localized hotspots typically forming near electrode tabs, which become more pronounced at high current densities and in larger‐format cells [311]. Guo et al. employed a distributed optical fiber sensing system based on optical frequency domain reflectometry (OFDR) to capture high‐resolution thermal profiles of commercial cylindrical cells [312]. With a spatial resolution of 3 mm, their measurements revealed axial and circumferential gradients that were previously undetected in lower‐resolution studies. During 1.5 C discharge, the maximum temperature (T_max_) consistently developed near the positive tab, with a surface temperature difference (ΔT) of 8.4°C across the cell.

Mitigating such localized heating requires careful optimization of the tab design. Waldmann et al. systematically examined the effect of the tab quantity in 26650‐type cells, demonstrating that increasing the number of cathode tabs from one to four substantially shortened the in‐plane current path through the Al current collector, thereby reducing the internal resistance (R_i_) [313]. As a result, both the overall temperature rise and T_max_ decreased with increasing tap number owing to lower ohmic heating (proportional to I^2^R). Additional tabs can also improve thermal coupling to external leads, further reducing the radial gradients, particularly under high‐C‐rate operation. This enhanced tab design mitigated the performance degradation and inhomogeneous aging during high‐rate cycling [313]. Complementing these experimental findings, Shen et al. simulated various tab configurations using an electrothermally coupled equivalent circuit network (ECN) model of an LG 2170 M50T cell [314]. Their simulations confirmed that increasing the tab number promoted a more uniform current distribution, suppressed localized heat generation, and moderated the temperature rise (Figure 7b). Importantly, they also identified the positive tab location as a critical factor governing thermal behavior.

#### 5.1.2 Pouch Cells

Pouch cells are widely regarded as the most mechanically vulnerable format because their soft laminate casing cannot withstand internal or external stresses such as swelling, compression, bending, or puncture [301]. Such stresses readily induce electrode misalignments, which may trigger ISCs or even catastrophic mechanical failures [315]. Bond et al. visualized electrode geometry changes in pouch cells using synchrotron‐based computed tomography (CT) [315]. Although their study employed a jelly roll electrode, stacked electrodes were generally even more prone to misalignment. CT imaging after overdischarge (Figure 7c) revealed pronounced misalignment in the flat jellyroll regions compared to the curved ones, and severe displacements in the electrode overhang areas. These results suggest that an insufficient stack pressure during manufacturing is strongly correlated with an increased deformation risk [316].

To mitigate the electrode deformation, many manufacturers have adopted Z‐stacking (or Z‐folding), in which a continuous separator is folded into a zigzag pattern with individually cut electrodes inserted between the folds, as shown in Figure 7d [317]. This design reduces the mechanical stress on the electrodes and separators, enhances interfacial adhesion, minimizes interlayer spacing, improves structural integrity, and ensures a more uniform pressure distribution [306, 318]. LG Energy Solution has further advanced this concept into an “advanced Z‐stacking” (AZS) process by incorporating heat lamination, which strengthens electrode–separator adhesion and further suppresses misalignment [319].

Despite these advances, pouch cells still face limitations in integrating intrinsic safety devices due to their external tab configuration. In practice, protection is usually provided externally via fusible links and thermistors. However, such safeguards respond too slowly to rapidly evolving thermal events [320]. To overcome this limitation, passive thermoresponsive materials have been embedded directly into separators, electrolytes, or electrodes [320, 321, 322, 323, 324]. Song et al. developed a scalable PTC layer comprising poly(3‐dodecylthiophene‐co‐3‐hexylthiophene‐co‐3‐triethylene glycol thiophene) (PDDHEO) as a thermoresponsive matrix and Super C as a conductive additive [323]. Under external stimulation, PF_6_^−^ ions de‐doped from the PDDHEO backbone disrupted the electronic conductivity and interrupted the current flow (Figure 7e). Notably, this design is compatible with roll‐to‐roll manufacturing, with a negligible impact on the energy density and rate performance. In impact tests of 3 Ah pouch cells, the PTC layer reduced explosion incidence by 53%, demonstrating both scalability and effectiveness.

#### 5.1.3 Prismatic Cells

Prismatic cells combine the advantages of both the pouch and cylindrical formats. Their rigid metallic casing facilitates the integration of safe devices and enables reliable sealing [301]. However, its prismatic architecture inherently limits heat dissipation, often leading to elevated heat generation and localized hotspots during operation [325]. Fan and Ma compared thermal management behavior in systems using cylindrical and prismatic battery cells, showing that cell format strongly affects heat dissipation and cooling efficiency under forced‐air cooling conditions [326]. In addition, Mohsen et al. developed a thermal model for a high‐energy prismatic lithium‐ion battery cell and module based on a dedicated thermal characterization methodology, demonstrating that heat accumulation can be concentrated in specific internal regions and exacerbate temperature non‐uniformity under high‐load conditions (Figure 7f) [327].

Additional simulations investigated the role of the cross‐sectional geometry by comparing laminated‐ and square‐type prismatic cells with equal volumes and capacities [328]. Laminated designs, owing to their larger surface area, showed lower T_max_ and smaller ΔT during discharge, though thermal uniformity remained limited (Figure 7g). Moreover, imperfect contact between the electrode layers and the rigid casing increases the susceptibility of prismatic cells to ISCs [329]. To mitigate these challenges, Z‐stacking technology has been increasingly implemented in prismatic designs. By reducing internal voids and enhancing interlayer cohesion, Z‐stacking improves structural uniformity, thereby supporting a higher energy density while simultaneously strengthening the resistance to mechanical deformation.

#### 5.1.4 Large‐Format Cell

Recent innovations exemplify how cell‐level design can simultaneously improve energy density and enhance safety. In 2022, Tesla initiated the deployment of 4680 cylindrical cells in Model Y, marking a transition from the 21 700 cells previously employed in Model 3 [330]. The 4680 cell features more than five times the volume and capacity, enabling economies of scale and greater energy density [331]. This cell adopts a “tabless” structure, more accurately described as a “continuous tab,” where hundreds of electrode tabs are generated through notching and folding of rectangular electrode [332]. Aluminum cathode tabs were grouped by ultrasonic welding and joined to an aluminum disk by laser welding. This design shortens the current pathways, ensuring uniform current distribution, reducing resistance, voltage drop, and local heating. On the anode side, copper tabs were directly connected to the bottom copper disk via laser welding. Therefore, the casing plays a role of the negative terminal, facilitating efficient current collection and enhanced heat dissipation [333]. ECN modeling confirmed that bottom cooling was significantly more effective than side cooling, particularly for large‐format cylindrical cells with thermally resistive radial layers [314, 334].

In 2020, BYD unveiled Blade cell, which is a large‐format prismatic cell that adopts a thermally stable LFP cathode. Its name is derived from its long shape and thin thickness (965 mm × 90 mm × 14 mm) [330]. Blade cell employs Z‐stacked electrodes fixed in place by laminated separator edges and a plastic rail to form a triple‐layer protective barrier. This barrier can mitigate ISCs by regulating the electrode misalignment during both manufacturing and cycling [335]. Furthermore, the Blade cell utilizes an Al housing instead of conventional steel, thereby reducing the overall weight and increasing the energy density. With a thermal conductivity of more than three times that of steel (180 vs. 60 W m^−1^ K^−1^), aluminum is well suited for side‐cooling systems [336, 337]. The thin, long geometry of the Blade further enhances heat dissipation along the cell surface, making it thermally and structurally optimized for safety [330, 338].

### 5.2 System‐Level Design

In this section, we review system‐level components and propose improvement strategies to enhance safety while also highlighting enabling technologies that support high integration for high‐energy‐density batteries.

#### 5.2.1 Cooling System

**FIGURE 8:**
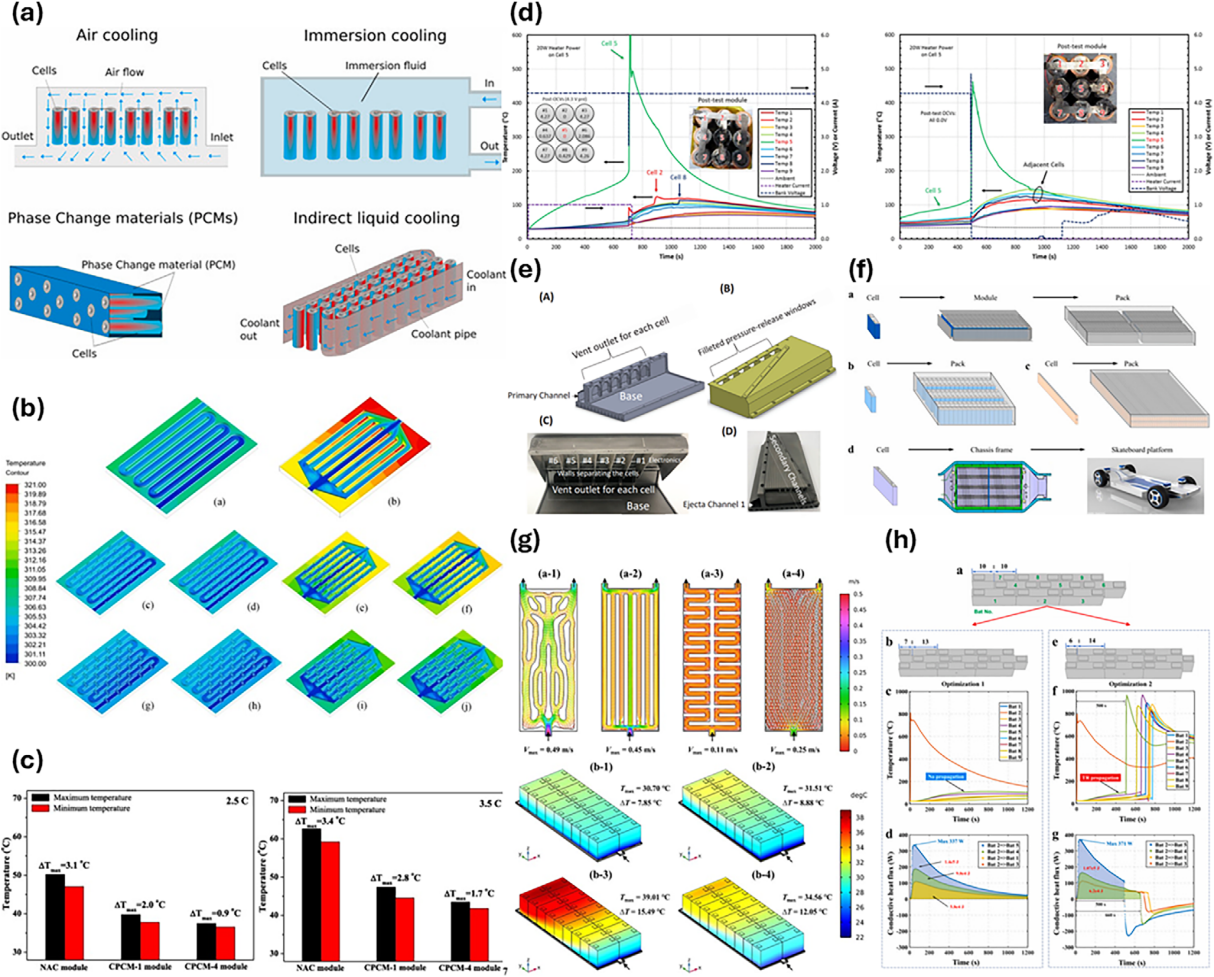
(a) Different thermal management systems. (Adapted from Charlotte, R. et al., 2022) (b) Temperature contours for SMCHS (left) and MMCHS (right) for different channel configurations. (Adapted from Nabeel, et al., 2021) (c) ΔT_max_ of the battery modules during the discharge process. (Adapted from Wangzhou, Y. et al., 2018) (d) Measured temperature response of each cell, connected with M‐type (left) and S‐type (right) tabs. (Adapted from Lopez, C.F. et al., 2015) (e) Designed battery module with six channels, featuring cell‐to‐cell separation walls, arched windows opening into channel 1 (open at one end), filleted pressure‐release windows on top, and individual top‐side channels (channel 2) for direct venting during thermal runaway. (Adapted from Srinivasan, B.G. et al., 2025) (f) Schematic representation of battery system architectures. (Adapted from Jin, C. et al., 2022) (g) (a) Velocity distributions and (b) module temperature distributions with different cold plates: (‐1) TCP‐2, (‐2) RCP, (‐3) SCP and (‐4) PCP. (Adapted from Luo, T. et al., 2024) (h) The optimization of the brick module. (Adapted from Jin, C. et al., 2022).

During battery operation, heat is inevitably generated due to both irreversible resistance losses and reversible entropy changes. Accordingly, thermal management has long been a critical element in commercial battery systems, and various cooling strategies have been developed (Figure 8a) [339]. Air cooling, which can be divided into natural and forced convection, is the most straightforward approach owing to its simplicity, low weight, and inherent safety. Natural ventilation relies on the ambient airflow, whereas forced convection employs cabin air circulation through heaters and evaporator coils. However, air cooling is inadequate under high‐power operation or fast‐charging conditions [340].

Liquid cooling has been increasingly adopted in grid‐scale battery systems because of its superior thermal conductivity and heat capacity [341]. Direct immersion in dielectric fluids provides excellent thermal performance. However, its implementation is limited by fire hazards, system complexity, and high costs. In contrast, indirect liquid cooling, which typically employs water/ethylene glycol mixtures, offers a more favorable balance of safety, efficiency, and manufacturability and is therefore the most widely adopted in practice [342, 343]. Within indirect systems, the coolant channel design, including the channel number, geometry (e.g., parallel versus serpentine), and flow rate, critically governs the heat transfer and pressure drop [344, 345]. Nabeel et al. compared serpentine mini‐channel heat sinks (SMCHS) with multi‐mini‐channel heat sinks (MMCHS) [344]. Although the SMCHS achieved ∼2.77 times higher Nusselt number, it also exhibited an approximately 39 times greater pressure drop. Among the pin‐fin designs, circular pin‐fins with grooves delivered the best balance of heat transfer and efficiency compared with semi‐circular, semi‐elliptical, and elliptical fins and grooves, as shown in Figure 8b. Cooling position is another crucial factor: in cylindrical 18650 cell modules, side cooling provided superior control of T_max_, whereas terminal cooling yielded smaller ΔT and more compact configurations [346]. However, as cell formats scale up from 21700 to 4680, side cooling becomes less effective, whereas tab cooling is important, although it should be noted that tab‐less architectures are essential to realize these benefits [334].

Heat pipes represent a promising passive cooling technology owing to their lightweight, compact structure, and efficient thermal transport via evaporation–condensation cycles. Yalong et al. optimized heat pipe systems by tailoring both the conductive pathways (e.g., height, arc spacing, and thickness) and heat sink geometry (e.g., fin number, spacing, and thickness), thereby enhancing the cooling capacity [347]. In particular, a helical heat pipe coupled with air cooling has been shown to increase the surface contact area, improve flexibility, and significantly boost heat dissipation. Nevertheless, challenges remain: intricate design, high cost, and leakage risks hinder large‐scale commercialization, particularly in bending or flexible structures. Further studies are required to overcome these limitations.

Phase‐change materials (PCMs), which are widely used in thermal energy storage, have also been explored for LIBs owing to their light weight, compactness, and high latent heat [348, 349]. During solid–liquid transitions, PCMs absorb or release substantial thermal energy, thus buffering cell temperature fluctuations. Ideal PCMs for LIBs should satisfy several criteria: (1) a phase transition temperature aligned with the operating range of batteries; (2) high latent and specific heat capacity; (3) high thermal conductivity; (4) low volume change during phase transition; (5) minimal supercooling and no phase separation; (6) non‐flammability and excellent thermal stability; (7) non‐corrosive and non‐toxic nature; and (8) low cost for practical deployment [350]. Paraffin satisfies many of these requirements but suffers from inherently low thermal conductivity, which leads to heat accumulation under repeated cycling and high‐rate discharge. This shortcoming can be addressed by incorporating thermally conductive additives such as carbon‐based materials or metal foams [351]. For example, expanded graphite (EG) markedly improves thermal conductivity, although it slightly reduces latent heat. At a 5 C discharge, the EG–paraffin composites effectively suppressed the temperature increase compared to pure paraffin [352].

However, the electrical conductivity of EG raises insulation concerns. To resolve this issue, electrically insulating yet thermally conductive additives such as silicon carbide (SiC) have been introduced [353]. An optimal composition of ∼15 wt.% SiC enhanced the resistivity and mechanical stability, whereas excessive SiC disrupted the EG network, diminishing the overall performance. The optimized composite achieved lower T_max_, reduced ΔT, and improved structural integrity, as shown in Figure 8c. Complementary strategies, including thickness control, fin integration, and conductivity‐gradient layering, have also been proposed to further improve the heat transfer in PCM systems [353].

#### 5.2.2 Flame‐Retardant Approach

At the module and pack levels, multiple cells are connected in series to increase the voltage and in parallel to enhance the capacity; however, such configurations remain vulnerable to thermal runaway propagation. The interconnect topology is particularly critical for determining thermal runaway propagation across the modules [354, 355]. Parallel cell modules not only enable more effective current balancing and higher capacity but also promote faster heat transfer than series‐cell modules. Experimental studies have demonstrated that increasing the degree of parallel connections reduces the thermal runaway onset temperature and accelerates propagation, particularly at the positive tab, which is subject to rapid heat accumulation and current imbalance [356]. Tab geometry can also modulate thermal runaway behavior. Lopez et al. compared the serpentine (S‐type) and branched (M‐type) tab designs in parallel configurations (Figure 8d) [166]. The S‐type tab geometry concentrated the current dumping into the failing trigger cell, causing elevated temperatures in the neighboring cells and a voltage collapse across the module. In contrast, the M‐type design distributes the current more evenly, which mitigates electrical drainage and delays propagation. These findings underscore the importance of both interconnect topology and tab configuration in suppressing thermal runaway propagation.

Incorporation of flame‐retardant barriers between cells has become a widely adopted strategy for interrupting thermal runaway pathways. A broad range of materials, including silicone rubber, mineral wool, hydrogels, aerogels, graphite composite sheets, and aluminum extrusions, have been explored for this purpose [357, 358]. Yang et al. developed a multilayer composite foam barrier consisting of polyurethane (PU) reinforced with flame‐retardant additives, combined with an intumescent layer and a firewall coating [359]. In this architecture, PU functioned as a structural support and flame‐retardant substrate, whereas the intumescent layer expanded upon heating to form a gas‐sealing heat‐blocking barrier. The firewall layer provides immediate thermal resistance. Nail penetration experiments and 3D thermal simulations confirmed that such multilayer barriers could completely block thermal propagation between adjacent cells.

Beyond conduction and convection, vented gaseous ejecta have been identified as the dominant contributors to thermal runaway propagation [360]. Srinivasan et al. reported that vented electrolyte solvents such as DMC can condense on neighboring cells and, upon ignition, transfer substantial heat that triggers cascading thermal runaway events [361]. To address this, their group proposed a vent‐management housing design (Figure 8e), incorporating three features: (1) a primary vent channel open at both ends, supplemented by secondary channels above each cell; (2) reinforced cell walls with arched windows; (3) improved housing material nickel‐plated Ultem 1010, capable of withstanding 1250°C and 100 psi. These features collectively alleviate vent clogging by solid ejecta, enable the rapid release of gaseous products, prevent the deposition of flammable vapors on adjacent cells, and maintain the structural integrity during thermal runaway events [362].

#### 5.2.3 Highly Integrated Battery System

In 2019, CATL officially unveiled cell‐to‐pack (CTP) technology, in which module frames were removed and cells were directly assembled into a pack (Figure 8f), achieving an energy density increase of approximately 15–20% [363]. Building on this concept, cell‐to‐chassis (CTC) integration, where cells are directly incorporated into the vehicle chassis, emerge and has been adopted by Tesla, BYD, CATL, and BMW [331, 364]. Compared with conventional battery systems, both CTP and CTC architectures introduce distinctive safety challenges: (1) the elimination of module frames and narrower cell spacing facilitate rapid heat transfer to adjacent cells; (2) bottom‐mounted cooling plates alone induce significant thermal gradients and hotspots; (3) single gas vent designs cause ejecta accumulation in poorly ventilated areas; (4) higher structural energy density accelerates heat release during failure; (5) the thermal conductivity of gap fillers varies under mechanical deformation, leading to BMS misestimation of actual cell temperatures; and (6) vertically stacked cells, or those located at the ends of coolant flow paths, exhibit thermal lag that promotes thermal runaway propagation [365, 366, 367].

Several system‐level safety strategies have been investigated to address this issue. Luo et al. designed a topology‐optimized cold plate for CTP systems that generated a flow channel distribution resembling the branching architecture of plant root systems [368]. Among the different inlet–outlet combinations, the single‐inlet dual‐outlet configuration was found to be optimal; a single inlet ensured a clear and controllable flow distribution, while dual outlets efficiently removed heat in both directions, mitigating accumulation at the flow path termini. The superior thermal performance of this root‐like channel design compared to the rectangular, serpentine, and pin–fin configurations is demonstrated in Figure 8g.

Jin et al. introduced a brick configuration in CTC systems in which prismatic cells are staggered to reduce the direct contact area between neighboring cells and enable heat dissipation across multiple adjacent cells [363]. Compared with the conventional in‐line arrangement, this architecture reduced peak heat flux by over 50%, and no thermal propagation was observed in either simulations or experiments. Optimization of the staggered area ratio further balanced safety and energy density, and thermal propagation occurred after 134.5 s in the in‐line layout and after 500 s in the 6:14 configuration; the 7:13 configuration completely suppressed propagation (Figure 8h), with an energy density penalty of less than 3%.

Building on this brick‐based concept, Wang et al. incorporated thermal insulation layers and forced convection as additional mitigation strategies [369]. Both methods were more effective in the brick layout than in the in‐line arrangement, reducing the required insulation thickness and cooling air velocity. However, the authors highlighted that small‐scale thermal runaway initiated by local insulation failure can lead to greater heat accumulation and accelerated propagation. To address this, they proposed a redundant insulation thickness as a necessary safeguard to enhance system‐level safety.

#### 5.2.4 Lifecycle Safety

5.2.4.1

Safety risks in LIBs do not end after first‐life operation, because retired cells and modules can retain substantial residual energy while containing degradation products and latent defects accumulated during use. In second‐life applications, cells with similar remaining capacities may still exhibit different aging histories, internal resistance, lithium plating behavior, gas accumulation, swelling, separator weakening, and thermal response. These hidden differences can lead to cell‐to‐cell imbalance, local heat generation, ISCs, and accelerated thermal runaway under renewed cycling or abnormal operating conditions [11, 370, 371]. Therefore, second‐life deployment requires systematic screening and classification based not only on SOH and capacity retention, but also on SOC, internal resistance, voltage relaxation, swelling, thermal response, and abuse tolerance.

End‐of‐life storage and transportation also introduce distinct safety challenges. Retired packs are often handled under uncertain SOC conditions and may have experienced mechanical damage, electrical isolation failure, moisture, temperature fluctuations, and long storage periods. Under these conditions, residual charge, damaged current collectors, weakened separators, or gas‐filled cells can trigger external or during handling. Practical end‐of‐life management should therefore include controlled discharge, terminal insulation, module‐level electrical isolation, mechanical protection, thermal monitoring, and fire‐resistant storage environments [371, 372]. These procedures are particularly important for heterogeneous battery batches, where cells with different chemistries, formats, manufacturers, and aging histories may be stored or transported together.

Recycling processes require dedicated safety protocols because disassembly, crushing, shredding, and material recovery can mechanically damage cells that still contain residual energy or reactive components. Incomplete discharge may induce short circuits during dismantling, while mechanical fragmentation can generate local heating, flammable gases, toxic gases, electrolyte vapor, and combustible dust [372, 373, 374]. Damaged cells or partially processed battery materials may also undergo delayed ignition or secondary fire during temporary storage. Therefore, recycling‐oriented safety management should combine reliable discharge verification, automated sorting, controlled atmosphere processing, ventilation, gas detection, fire suppression, and safe storage of intermediate products.

Incorporating lifecycle considerations extends the materials‐to‐systems framework from battery operation to second‐life use, end‐of‐life handling, and recycling. From a cascade‐aware perspective, lifecycle safety can be regarded as an extension of system‐level safety because aging history, residual energy, mechanical damage, and processing conditions can act as delayed triggers for the same thermal runaway pathways discussed in operating cells and packs. Thus, future safety protocols should integrate first‐life usage records, second‐life qualifications, end‐of‐life discharge control, transport safety, and recycling‐process monitoring into a continuous battery safety management framework.

## 6 Battery Management Systems (BMS) for Safety Enhancement

A BMS is a critical electronic system that oversees battery operation by continuously monitoring key parameters, such as voltage, current, and temperature, while preventing overcharging, overdischarging, and overcurrent to ensure safe operation. BMS architectures typically connect to all individual cells and pack components and operate through a three‐layer hierarchy: physical, prediction, and control layers [375, 376]. The physical layer collects real‐time data on cell voltage, temperature, and other operational metrics by employing distributed fiber‐optic thermometers, voltage sensors, and Micro‐Electro‐Mechanical System (MEMS)‐based gas sensors, which utilize these measurements to estimate the state of individual cells, modules, and the full pack, predicting critical parameters such as the SOC and SOH. The control layer implements cell balancing, power limitation, and thermal management strategies based on the estimations from the prediction layer [377].

Research on BMS has primarily focused on two areas, hardware‐based sensing technologies and algorithmic state estimation methods, to improve the detection and prediction of hazardous conditions [378, 379, 380, 381, 382, 383, 384, 385, 386, 387, 388, 389, 390, 391, 392, 393, 394, 395, 396]. Early BMS implementations relied on simple protection circuit boards (PCBs) to monitor overcharge, overdischarge, and overcurrent events [397]. However, PCB‐based methods suffer from detection delays of up to 1.2 s per cell and may experience critical instability during thermal runaway, rendering them inadequate for high‐risk conditions [398]. To overcome these limitations, modern BMS designs are increasingly integrating sensors into the physical layer, providing more precise measurements of current, voltage, and temperature to the prediction layer [399].

The prediction layer faces additional challenges due to the complex multi‐physics nature of battery failure. For example, thermal runaway arises from coupled phenomena including SEI decomposition, electrolyte gas evolution, and ISCs, which cannot be reliably captured using a single method [400]. To address this complexity, recent studies have incorporated artificial intelligence (AI) and machine learning into the prediction layer, enabling rapid and robust detection of potential safety issues [401]. Physics‐based models have also been employed to enable a more accurate prediction of intricate behaviors such as the nonlinear voltage responses observed in the two‐phase plateaus of LFP batteries [402]. This section reviews the latest developments in BMS technologies, emphasizing the advances in sensing, predictive modeling, and control strategies that collectively enhance battery safety at the cell, module, and pack levels.

### 6.1 Real‐Time Monitoring and Control through Sensing

Optical Fiber Bragg Grating (FBG) sensors have emerged as highly promising tools for monitoring battery health, owing to their low cost, compact size, and exceptional temperature‐sensing capabilities. An FBG sensor consists of a short segment of a single‐mode optical fiber with a periodically modulated refractive index in the core engineered through photoinduction. This structure reflects specific wavelengths of light, allowing precise measurements of the temperature, pressure, and strain. In addition, FBG sensors are particularly suitable for in‐cell applications because they are immune to electromagnetic interference, non‐conductive, and chemically inert [378].

Yang et al. first applied FBG sensors to LIBs to monitor the temperature variations [378]. An array of seven FBG sensors was attached to the surface of the coin cell to track the temperatures of both the anode and cathode. Their sensors achieved a temperature resolution of 0.1°C and a temperature sensitivity of 10 pm °C^−1^, with a wavelength resolution of 1 pm at a sampling rate of 2 Hz. They demonstrated linear responses under varying current, overcharge, and external short circuit conditions. Expanding this approach to larger formats, Novais et al. applied FBG sensors to pouch cells and monitored the temperature changes at the tab‐electrode junctions and electrochemically active regions during experiments at different C‐rates [379]. They observed that the temperature variations were directly correlated with the applied current slopes, indicating that Li ions migrated inside the cell to establish a concentration gradient, which generated heat as a function of the applied current. Fleming et al. applied FBG sensors to cylindrical cells to measure the temperatures of both the core and outer surfaces [380]. They observed notable temperature differences, increasing up to 6°C during discharge and 3 °C during charging. Electrochemical impedance spectroscopy (EIS) and high‐resolution CT analyses confirmed that these gradients were intrinsic to the cell rather than a limitation of the sensors, highlighting that both the core and surface temperatures need to be monitored independently for accurate thermal assessment.

FBG sensors have also been used to detect internal chemical reactions such as SEI decomposition and gasification, which are key triggers of thermal runaway. Huang et al. reported the first real‐time observation of SEI formation using FBG sensors, which enabled the simultaneous measurement of temperature and pressure within electrochemical cells [381]. To differentiate the contributions of the temperature and pressure to the wavelength shifts of the reflected peaks, they combined single‐mode optical fibers (SMF‐FBGs) with micro‐structured optical fibers (MOF‐FBGs). By analyzing wavelength shifts, they distinguished temperature‐ and pressure‐induced contributions, capturing SEI formation through characteristic temperature (45–55 °C) and pressure peaks. To further quantify SEI formation, the authors measured the heat generated inside the battery during the first formation cycle using computational calorimetry and applied a thermal circuit model that decomposes the heat into irreversible heat owing to overpotential and reversible heat corresponding to entropy change during charge and discharge. These approaches enable the detection of cell‐temperature gradients and the conversion of thermal events into quantifiable heat events, enabling the tracking of SEI formation and cell lifetime.

Further advancements include the use of tilted‐FBG sensors (TFBGs) to assess the electrolyte turbidity and chemical reaction pathways of SEI formation [382]. TFBGs measure refractive index changes, predict electrolyte decomposition, and quantify particulate concentrations via cladding‐mode amplitude reduction, which is indicative of turbidity. Generalizing this approach across electrolyte formulations revealed strong correlations between capacity fade and two electrolyte‐related metrics: refractive index variation (Pearson r ≈ 0.97–1.00), which reflects changes in electrolyte composition, and normalized peak‐to‐peak amplitude loss (r ≈ 0.73–0.85), associated with particulate accumulation and turbidity. These findings demonstrate that TFBG‐derived optical signals can serve as sensitive, non‐destructive indicators of electrolyte degradation, enabling early detection of solvent decomposition and particulate formation before significant capacity loss.

Despite these advancements, the accurate detection of pressure peaks after the first cycle remains challenging due to SEI formation [381]. Therefore, instantaneous measurement of both pressure and temperature is essential for assessing thermal runaway beyond the initial cycle. To address this need, Nascimento et al. introduced a hybrid FBG–Fabry–Perot interferometer (FPI) approach [383]. The FPI is specifically designed to measure the pressure, mechanical deformation, and minute changes in the distance between two reflective surfaces within a cavity. Operating on a principle analogous to FBG sensing, the FPI was embedded in the top, middle, and bottom regions of the lithium‐ion pouch cells, enabling in situ monitoring of internal strain and temperature variations. This configuration establishes quantitative relationships between strain and temperature because elevated temperatures facilitate Li^+^ migration, resulting in increased strain.

**FIGURE 9:**
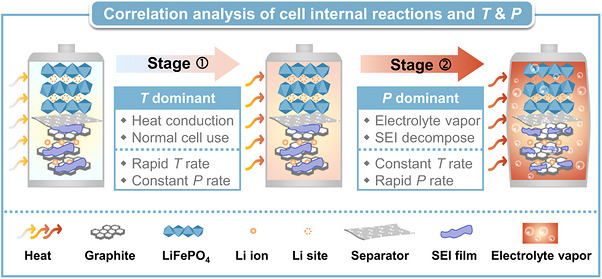
Characteristic two‐stage variations of pressure and temperature observed during irreversible reactions in the battery (Adapted from Mei, W. et al., 2023).

To enable sensors to remain operational and retain their measurement capabilities during and after thermal runaway, Mei et al. developed a multifunctional optical fiber sensor integrating a femtosecond‐laser‐inscribed FBG with an open‐cavity FPI [384]. Traditional sensors often detect gases released from safety venting or voltage drops induced by ISCs late to prevent irreversible degradation. To enable the early detection of irreversible reactions, the authors proposed monitoring the onset of gasification and SEI decomposition by analyzing slope changes in the differential curves of temperature and pressure. This method allows for the predefinition of safety warning thresholds by identifying the transition between reversible and irreversible reactions. When irreversible processes occur, the time derivatives of the temperature and pressure exhibit two distinct stages: an initial stage, in which the temperature rises rapidly at a nearly constant pressure, followed by a second stage, in which the temperature continues to increase steadily alongside an accelerated pressure rise (Figure 9). The transition between these stages may correspond to irreversible SEI decomposition, typically occurring around 70–80°C, providing a practical early warning indicator for impending thermal runaway.

In recent years, with the increasing adoption of lithium metal anodes in all‐solid‐state batteries, real‐time monitoring of Li plating has become critical. In this context, substantial efforts have been made to visualize Li plating using ultrasound. While previous studies employing FBG and FPI sensors have primarily predicted the battery status through temperature and pressure measurements, they have not fully addressed spatially varying variables such as strain. In contrast, ultrasound enables detailed mapping of the internal parameters within the cell, allowing precise identification of locations where Li plating and associated risk events occur. Wasylowski et al. demonstrated that the reflection signal intensity increased by approximately 30% owing to changes in the acoustic impedance at the cathode surface during Li plating [402]. By segmenting the signal into bandwidth‐defined time gates and analyzing each individually, the authors achieved a layer‐level resolution that was sufficient for distinguishing adjacent components, including electrodes, current collectors, and separators. This differentiation allows for the precise localization of Li‐plating events. Integrating the ultrasound signal within each gate generates scalar values that can be visualized as a color‐mapped image, which, when repeated across the cell, produces a comprehensive spatial profile that can be directly compared with post‐mortem examinations to confirm the Li plating sites.

### 6.2 AI‐Driven Prognostics and Diagnostics

With rapid advancements in sensor technologies, real‐time data acquisition and battery health estimation have attained unprecedented levels of sophistication. The operational conditions of a battery, including its degradation state and remaining lifespan, can vary considerably depending on the user behavior, environmental factors, and external conditions. The accurate prediction of these outcomes requires the comprehensive consideration of multiple variables, an approach that can be significantly enhanced using AI models. Early studies employed AI models such as Equivalent Circuit Models (ECMs), Pseudo‐2D (P2D), and thermal models to predict battery health [403, 404, 405, 406, 407, 408, 409]. Among these, ECMs have been particularly favored due to their simplicity, stability, and interpretability [410, 411]. ECMs are lumped‐parameter models comprising networks of passive components, including resistors, capacitors, and voltage sources, which estimate cell terminal voltage from applied charge or discharge currents. Nonlinear discrepancies between the sensor measurements and model predictions were managed using Kalman filters (KF) or Extended Kalman filters (EKF) to update the SOC and SOH [400, 412, 413, 414].

However, the resistance and capacitance values within ECMs fluctuate with temperature and battery degradation, affecting the estimation of internal resistance and complicating SOC prediction, particularly at low temperatures, where internal polarization and potential two‐phase coexistence can significantly reduce accuracy [415]. The estimation errors are further exacerbated near the open‐circuit voltage (OCV) region, where low‐order Taylor expansions fail to fully describe the nonlinear voltage behavior. Moreover, the standard KF and EKF algorithms assume Gaussian noise distributions, which only approximate real‐world sensor noise and system dynamics, thereby limiting their robustness [416]. To overcome these challenges, Zhang et al. developed a hybrid Backpropagation Neural Network‐Extended Kalman Filter (BPNN‐EKF) algorithm [416]. This approach leveraged neural networks trained on data from LFP batteries under varying conditions, and feature selection was performed using the Pearson correlation coefficient. BPNN models capture the nonlinear relationships between input variables and outputs through activation functions, with node parameters updated across multiple layers using backpropagation. When combined with the EKF, this algorithm can successfully correct prediction errors and effectively model nonlinear interactions among the current, voltage, and temperature. This hybrid BPNN‐EKF approach effectively overcomes the key limitations of conventional SOC estimation methods. Traditional EKF‐based techniques often rely on low‐order Taylor series expansions, which result in higher estimation errors in regions where the voltage is minimally sensitive to the SOC. Moreover, the Gaussian noise assumption inherent to the EKF does not fully capture the stochastic nature of real‐world sensor measurements and dynamic system behavior, thus reducing the robustness under variable operating conditions. By addressing these challenges, the BPNN‐EKF algorithm achieves root‐mean‐square errors of 3.98% at −20°C, 3.62% at 10°C, and 1.68% at 35°C, demonstrating markedly improved accuracy and robustness compared to conventional EKF or standalone data‐driven approaches, particularly under low‐temperature and high‐dynamic load scenarios.

**FIGURE 10:**
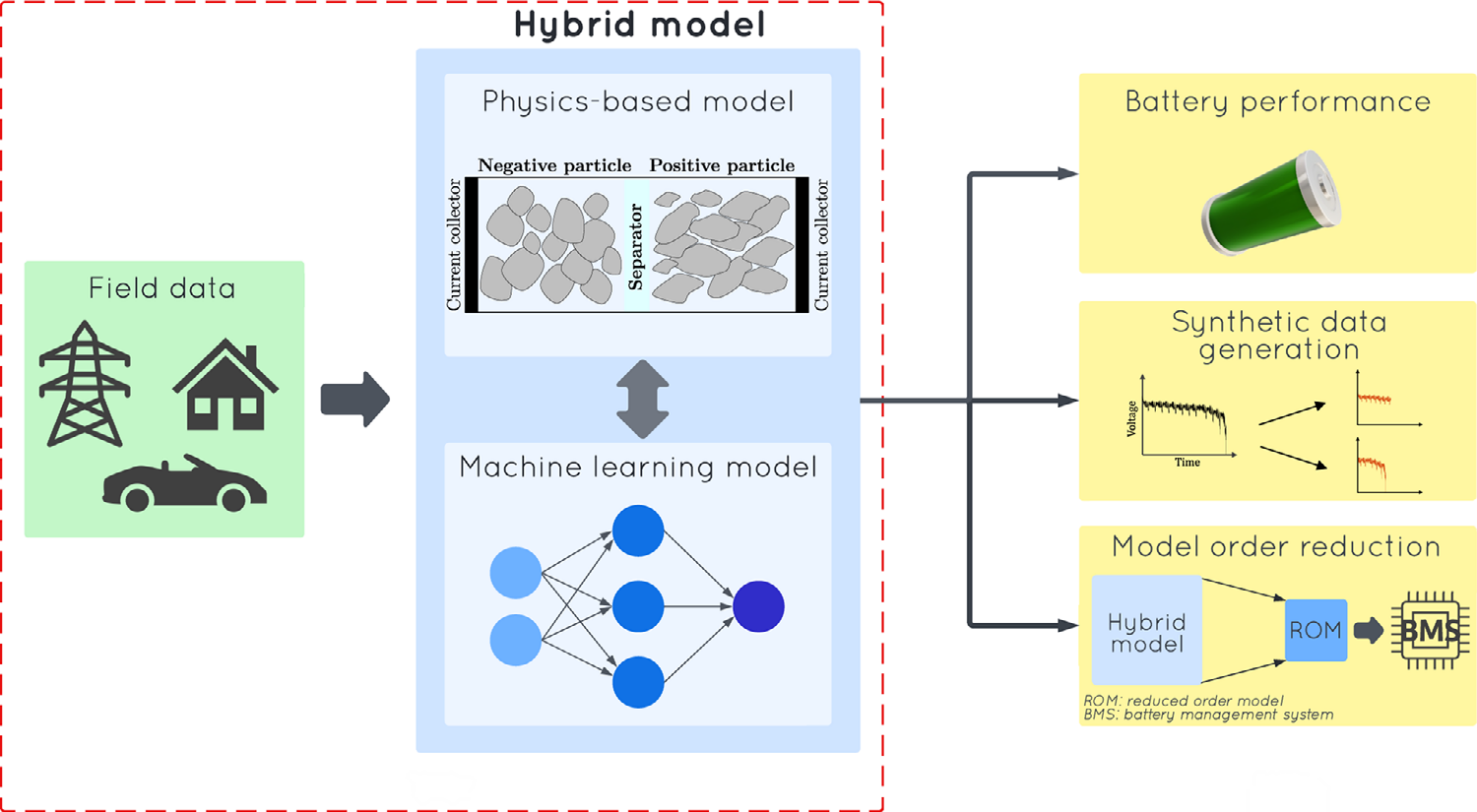
Schematic representation of hybrid models that combines physics‐based models and machine learning models (Adapted from Pozzato, G et al., 2024).

To overcome the inherent limitations of machine‐learning models, which are highly dependent on the quantity and quality of training data, extensive research has focused on hybrid approaches that integrate data‐driven methods with physics‐based models [388]. Figure 10 provides a schematic overview of hybrid systems that couple enhanced single‐particle models with mass conservation or thermodynamic formulations. These hybrid approaches can be implemented in two main ways: by using machine learning to augment physics‐based models or by leveraging physical models to generate training data for machine learning algorithms. For example, Tian et al. developed a Long Short‐Term Memory (LSTM) deep neural network (DNN) augmented with physical models [389]. This approach incorporates an online ECM that decomposes the terminal voltage into constituent components in real time using a Kalman filter based on the Thevenin equivalent circuit and Monte Carlo dropout. These physics‐based components address the common shortcomings of DNNs, including performance degradation from limited training data and neglecting temporal dependencies. By providing physically meaningful voltage features that reflect the internal battery dynamics, online ECM can enhance the input representation and improve generalization across variable operating conditions. Concurrently, the Monte Carlo dropout enables the DNN to estimate the SOC with associated uncertainties, which are fused with one‐step ampere‐hour predictions using the Kalman filter. This fusion mitigates overfitting and ensures temporally consistent uncertainty‐aware SOC estimations. The resulting hybrid framework reduced the root‐mean‐square error (RMSE) of the baseline DNN from 2.51% to 1.85% and reduced the mean absolute error (MAE) of SOC estimation from 9.54% to 3.35%. Similarly, Pozzato et al. combined a machine learning model trained on the voltage hysteresis phenomena of LFP batteries with a physics‐based average core‐shell enhanced single‐particle model (ESPM) [387]. This integration preserves the fundamental physical principles of lithium‐ion transport and insertion by explicitly modeling lithium diffusion, electrolyte mass transport, and charge conservation. ESPM captures phase‐separation dynamics in LFP particles through a core–shell representation that reflects the evolution of Li‐rich and Li‐poor phases during intercalation, ensuring that the modeling outcomes accurately mirror the experimental thermodynamic and kinetic conditions. By incorporating these mechanisms, the hybrid model reproduces the pseudo‐hysteresis observed in LFP, which originates from the path‐dependent lithium distributions within individual particles during charge and discharge cycles. Moreover, by training on a low‐dimensional dataset, the approach reduced computational costs while achieving a 95% reduction in training error and an 83% reduction in testing error for voltage predictions at high states of charge, where hysteresis effects are most pronounced.

On the thermal side, Pang et al. developed a physics‐informed neural network (PINN) to predict heat generation in LIBs [390]. Grounded in a single‐particle model that applies the first law of thermodynamics, the heat generation is attributed to ohmic losses, electrochemical reaction heat, and entropic contributions. Surface concentrations from the single‐particle model were input into a bidirectional LSTM network with hyperparameters optimized using Bayesian methods. By embedding energy conservation within the training process, this approach achieved higher accuracy and generalization under dynamic operating conditions, with a mean absolute error of 0.542 kW m^−3^ and a root‐mean‐square error of 1.428 kW m^−3^ during standardized light vehicle tests.

Recent developments in Digital Twin (DT) technology aim to bridge the gap between model predictions and real‐world battery behaviors. A DT is a high‐fidelity virtual replica that mirrors both the physical geometry and dynamic phenomena of a system [391]. Song et al. developed a digital twin‐driven ECM model for a single NMC811 particle, enabling the real‐time simulation of structural deformation induced by Li intercalation [392]. The model employs a combination of optical microscopy (OM) and scanning electron microscopy (SEM) to reconstruct detailed three‐dimensional representations of pore structures and cracks within secondary particles. To resolve pore morphologies and interparticle cracks at the nanoscale, challenging to observe with conventional SEM, focused ion beam (FIB) milling was combined with SEM to generate 60 cross‐sectional images at 100 nm intervals, which were subsequently reconstructed into a precise 3D structure. This high‐resolution modeling facilitates the analysis of the operando voltage profiles, overpotentials, Li concentration distributions, and von Mises stresses. By adjusting parameters such as primary and secondary particle sizes within the digital twin‐based ECM, the study revealed that capacity retention at high C‐rates is predominantly determined by the secondary particle dimensions, which govern Li diffusion lengths. Moreover, although the conventional single‐particle model does not inherently account for the internal stresses associated with crack initiation, the authors successfully identified potential crack formation by imposing boundary constraints to simulate stress concentrations.

Park et al. extended DT approaches to predict thermal runaway, particularly under extreme conditions, such as a 10 C discharge rate [393]. Traditional lumped models that lack internal structural resolution have limited accuracy in such scenarios [394, 395, 396]. In contrast, the authors implemented a microstructure‐resolved thermo‐electrochemical DT framework that reconstructed the three‐dimensional microstructure of pouch cells and integrated it into a full‐cell‐to‐module‐scale simulation. This framework captures the spatially heterogeneous heat generation and temperature distributions by explicitly resolving the structural heterogeneity overlooked in conventional lumped models. Under a high rate 10 C discharge, the DT model predicted a peak internal temperature of 137.2°C, markedly exceeding the 123.9°C estimated by the conventional model. This divergence underscores the ability of DTs to detect localized overheating and non‐uniform thermal propagation. Furthermore, the model provides spatially resolved internal temperature maps, enabling the identification of thermal gradients and hotspots that serve as critical indicators of thermal runaway risk under abusive or high‐load conditions.

## 7 A Comprehensive Perspective on Battery Safety

As discussed in this review, thermal runaway in LIBs is not the consequence of a single component, but is a complex, multifactorial phenomenon arising from coupled thermal and chemical interactions among the cathode, anode, separator, electrolyte, and other internal interfaces. Crucially, thermal runaway does not terminate with the decomposition of a specific material; rather, it propagates through a cascade of sequential reactions, each of which triggers the next stage. The onset conditions and propagation pathways vary markedly with the cell geometry (e.g., pouch, cylindrical, and prismatic), assembly structure, and chemistry (e.g., NMC, LFP, and Li‐metal), and are further modulated by external stresses such as overcharging, mechanical impact, or localized heating. In response to this complexity, a wide spectrum of safety strategies‐ranging from material‐level innovations to structural engineering and operational control technologies, including BMS and AI‐based diagnostics, have been actively explored.

Despite these efforts, current approaches frequently fail to achieve comprehensive system‐level suppression of thermal runaway. Many studies have focused on enhancing the safety of individual materials or under specific thermal thresholds while overlooking the full cascade of interdependent failure pathways. For example, although a flame‐retardant electrolyte additive may delay ignition, it cannot prevent subsequent cathode decomposition, oxygen release, or inter‐cell heat transfer, all of which can escalate the event. Interrupting a single failure mode rarely guarantees full thermal stability because the nonlinear and interfacial dynamics across materials, layers, and thermal zones enable runaway to progress beyond the initial trigger.

At the early stage of LIB commercialization, safety was recognized as an optional performance factor, especially for small‐sized cells. However, as large‐scale LIB stacking systems have been deployed from kWh to MWh, the safety issue has become increasingly critical due to the risk of catastrophic fire accidents. While safety regulations have promoted the efficient design of battery packs and modules with densely aligned cells to achieve high energy density, they have also intensified the need for more reliable safety strategies. Consequently, the development of safer LIBs will play a key role in improving the performance and reliability of large‐format battery systems. Although advances in materials and manufacturing technologies are expected to further reduce the production cost of LIBs, the economic and societal costs associated with safety accidents will not decrease accordingly. Therefore, safety technologies are expected to receive greater emphasis in the next stage of LIB development than in the past. To make this perspective actionable, LIB safety should be organized according to the stage of failure evolution, the measurable indicators available at each stage, and the intervention that can still interrupt the failure cascade. In this framework, thermal runaway is not treated as a single endpoint defined only by fire or explosion. Instead, it is evaluated as a progressive sequence involving aging‐related degradation, local heat accumulation, interfacial reactions, separator failure, self‐heating acceleration, and cell‐to‐cell propagation. Table 6 summarizes representative indicators, warning criteria, intervention targets, and design guidelines that can translate the cascade‐aware concept into practical safety design.

**TABLE 6:** Stage‐specific indicators and intervention criteria for a cascade‐aware LIB safety framework [93, 95, 96, 97, 98, 99, 134, 135, 157, 160, 164, 168, 169].

**Failure stage**	**Representative indicators**	**Risk criteria or warning signs**	**Intervention target**	**Actionable design guideline**
Normal operation and aging	SOH, capacity retention, internal resistance, charge‐transfer impedance, core–surface temperature gradient	Continuous impedance growth, enlarged thermal gradient, abnormal voltage relaxation, accelerated capacity fade, and delayed surface detection of internal heat accumulation	Prevent latent degradation from becoming a safety trigger	Use lifetime‐aware BMS, update SOC and SOH operating limits, detect abnormal impedance rise, combine surface temperature with voltage and impedance diagnostics
Early abuse or localized heating	Local temperature, tab temperature, voltage deviation, current imbalance, thermal sensitivity coefficient	Localized heating near tabs or heterogeneous electrodes, rapid temperature rise at high SOC, abnormal voltage fluctuation, and shortened runaway onset under concentrated heating	Suppress hot‐spot formation before global thermal instability	Improve current‐collector and tab design, optimize electrode homogeneity, place sensors near thermal hot spots, apply current limitations during abnormal heating
Interfacial reaction stage	SEI decomposition, gas evolution, self‐heating onset, anode–electrolyte heat release	Latent stage below ∼100 °C and intermediate stage of ∼100–150 °C, where SEI and anode‐related exothermic reactions can dominate under insufficient cooling	Stabilize the anode–electrolyte interface and delay autocatalytic reactions	Use robust SEI‐forming additives, suppress lithium plating, apply thermally stable electrolytes, reduce local overpotential and internal resistance
Separator failure stage	Separator shrinkage, impedance collapse, internal short circuit, voltage instability	Polyolefin separator shrinkage at ∼120–130 °C and melting at ∼160–170 °C, with impedance reduction and internal short formation near separator contraction	Maintain electrode separation and prevent internal short circuits	Use ceramic‐coated, hybrid, or shutdown separators, improve dimensional stability, reinforce separator mechanical integrity, design separators with high‐temperature tolerance
Runaway acceleration stage	Self‐heating rate, voltage collapse, pressure rise, gas generation, peak temperature	ARC self‐heating onset when dT/dt exceeds ∼0.02 °C min^−1^, full runaway when dT/dt exceeds 10 °C min^−1^, and rapid acceleration to >30 °C min^−1^ near 200–220 °C	Interrupt self‐sustaining heat generation and reduce pressure buildup	Trigger active cooling, electrical isolation, venting control, pressure relief, flame‐retardant electrolyte response, and module‐level emergency shutdown
Cell‐to‐cell propagation stage	Adjacent‐cell temperature, heat flux, transferred energy, propagation delay time, gas jet direction	Adjacent‐cell temperature approaching ∼200 °C, transferred energy of ∼10–14 kJ under tight spacing, and insufficient spacing below propagation‐resistant design ranges	Prevent single‐cell failure from becoming module or pack failure	Increase cell spacing where possible, insert thermal barriers, design vent paths, prevent gas accumulation, isolate failed cells, combine propagation‐resistant layout with BMS‐level fault response

**FIGURE 11:**
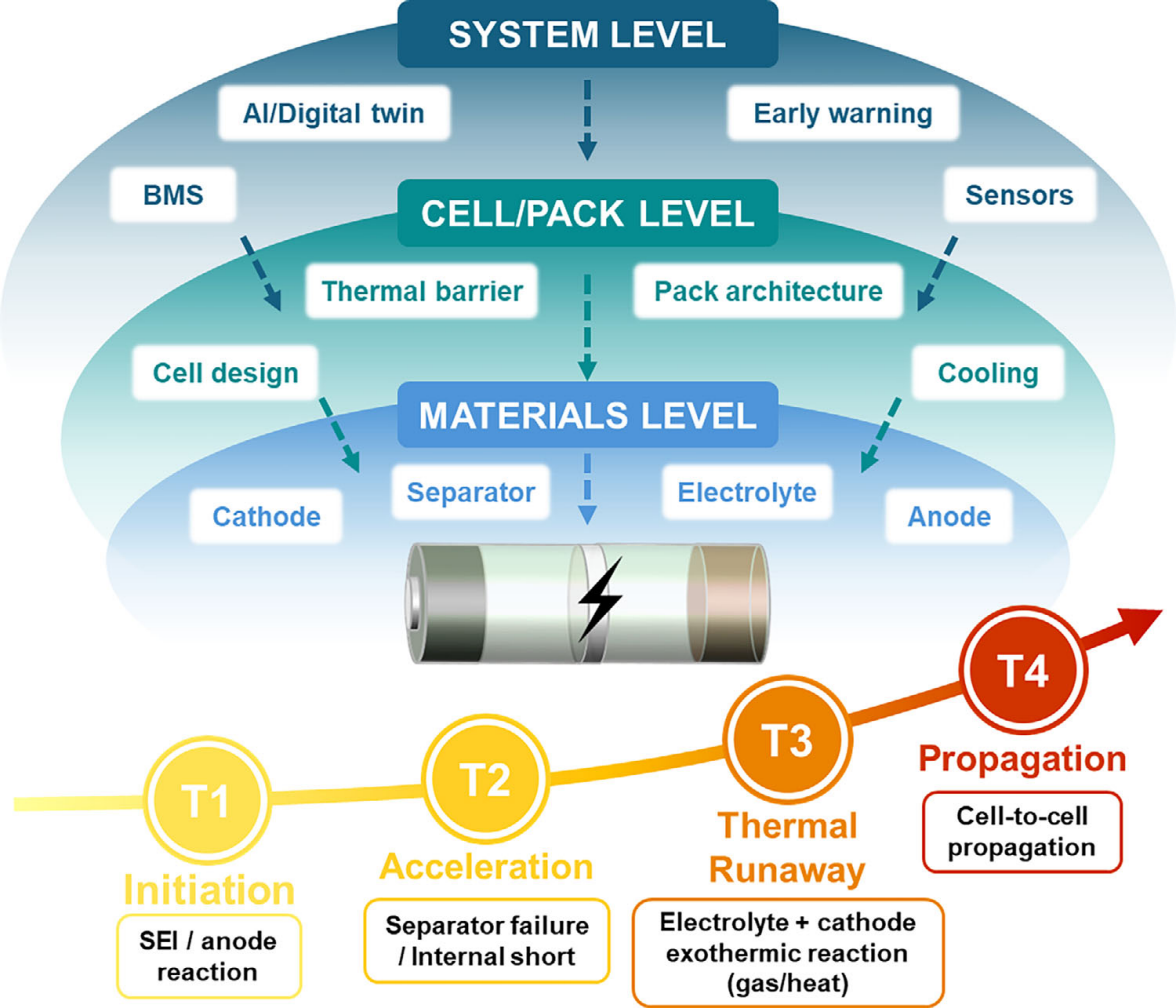
Summary schematic of the cascade‐aware safety framework for LIBs.

The overall cascade‐aware safety concept is summarized in Figure 11, which links the T_1_–T_4_ thermal runaway sequence with materials‐, cell/pack‐, and system‐level intervention strategies. This stage‐specific framework provides a practical decision pathway for safety design. During normal operation and early abuse, the priority is prediction and trigger suppression through impedance tracking, thermal‐gradient monitoring, voltage‐anomaly detection, and current limitation. During interfacial reactions and separator failure, the focus shifts to material‐level stabilization, including robust SEI chemistry, Li‐plating suppression, nonflammable electrolytes, and thermally stable separators. Once self‐heating acceleration begins, the effective intervention window becomes narrower, making system‐level actions such as active cooling, venting, electrical isolation, and emergency shutdown essential. At the propagation stage, the design objective shifts from protecting the failed cell to preventing heat, gas, flame, and ejecta from reaching neighboring cells. Accordingly, cascade‐aware safety design can be implemented through three practical steps. First, cell‐specific warning thresholds should be calibrated using measurable parameters such as onset temperature, self‐heating rate (dT/dt), impedance growth, gas evolution, pressure rise, voltage collapse, and adjacent‐cell temperature. Second, these parameters should be mapped to stage‐specific interventions, ranging from materials‐level stabilization to pack‐level isolation. Third, the calibrated thresholds should be embedded into BMS and digital‐twin platforms so that risk levels can be updated according to aging state, SOC, operating condition, and module geometry. This protocol converts the cascade‐aware safety framework from a conceptual description into an engineering approach that connects measurable failure indicators with actionable safety responses. Beyond technical implementation, progress in LIB safety must also be supported by regulatory, environmental, and industrial frameworks. Commercial deployment of high‐reliability batteries requires standardized thermal runaway tests, harmonized safety evaluation protocols, and the adoption of environmentally benign and recyclable safety materials. Lifecycle safety protocols for second‐life screening, end‐of‐life storage, transportation, safe discharge, recycling, and secondary fire prevention are also essential for ensuring long‐term sustainability.

Ultimately, safety is no longer a peripheral consideration, but a prerequisite for the societal acceptance of high‐energy storage systems. Establishing quantitative, stage‐specific, and cascade‐aware safety foundations will support the development of LIBs that are not only higher in energy density, but also more predictable, controllable, and reliable across their full lifecycle.

## Author Contributions

**Jin Hyeok Yang**: conceptualization, investigation, writing – original draft. **Jae Yoon Sung**: writing – original draft, investigation. **Hynji Kweon**: investigation, writing – original draft. **Seohyun Kim**: investigation, writing – original draft. **Byunghoon Kim**: writing – review and editing, supervision. **Jongsoon Kim**: writing – review and editing, supervision. **Junyoung Mun**: writing – review and editing, supervision. **Jung Ho Kim**: writing – review and editing, supervision. **Ki Jae Kim**: writing – review and editing, supervision, conceptualization.

## Funding

This work was supported by the National Research Foundation (NRF) of Korea grant funded by the Korean government (MSIT) (No. RS‐2025–25441255) and by the National Research Council of Science & Technology (NST) grant by the Korea government (MSIT) (No. GTL25091‐200).

## Conflicts of Interest

The authors declare no conflicts of interest.

## Data Availability

Data supporting the findings of this study are available from the corresponding author upon request.
